# Spectroscopic Tracking of Size Selectivity in Crown Ethers: A Unified Molecular Picture of Encapsulation

**DOI:** 10.1021/jacs.6c05693

**Published:** 2026-08-05

**Authors:** Ryu Sakuma, Keisuke Hirata, James M. Lisy, Masaaki Fujii, Shun-Ichi Ishiuchi

**Affiliations:** † Department of Chemistry, School of Science, 724580 Institute of Science Tokyo, 2-12-1 Ookayama, Meguro-Ku, Tokyo 152-8550, Japan; ‡ Laboratory for Chemistry and Life Science, Institute of Integrative Research, Institute of Science Tokyo, 4259 Nagatsuta-cho, Midori-Ku, Yokohama 226-8503, Japan; § Research and Development Initiative, Chuo University, 1-13-27 Kasuga, Bunkyo-ku, Tokyo 112-8551, Japan; ∥ Institute of Integrative Research (IIR), Institute of Science Tokyo, Nagatsuta-cho, Midori-Ku, Yokohama 226-8503, Japan; ⊥ Department of Chemistry, University of Illinois at Urbana−Champaign, Urbana, Illinois 61801, United States

## Abstract

Crown ethers exhibit pronounced ion selectivity and have long provided an excellent platform in supramolecular chemistry. This selectivity has been rationalized by a match between the ionic radius and the nominal cavity size of the crown ether, largely driven by the success of the K^+^18-crown-6 system. However, this concept omits the role of conformational flexibility of the crown ethers and fails to directly connect binding structures with ion selectivity. To gain molecular-level insight into the intrinsic binding structures of crown ethers with alkali metal ions, we present a systematic study of crown ethers with varying ring sizes using cryogenic gas-phase infrared spectroscopy in conjunction with anharmonic analysis of the CH stretch region. The CH stretch region serves as a sensitive probe of ion-binding structures and interactions, enabling definitive structural assignments of Li^+^–Cs^+^ complexes across different crown ethers. Our results reveal that the intrinsic binding structures vary systematically with both ionic radius and crown ring size. It appears that successful selectivity for a specific ion-crown ether system depends on two factors: the ability of the crown ether to encapsulate the ion, and having a configuration where the ion is equidistant from the oxygen atoms within a plane formed by the etheric oxygens. While encapsulation of Li^+^, Na^+^ and K^+^ is achieved by 18-crown-6, only K^+^18-crown-6 has the oxygen distances uniformly positioned about this plane, resulting in truly selective encapsulation. For 12-crown-4 and 15-crown-5, the most favorable structures, involving Li^+^ and Na^+^, respectively, are unable to achieve selective encapsulation, as the ions are positioned at varying distances with respect to the plane of the etheric oxygens. These distinctions can be traced to the differing conformational flexibility of the crown ethers.

## Graphical abstract

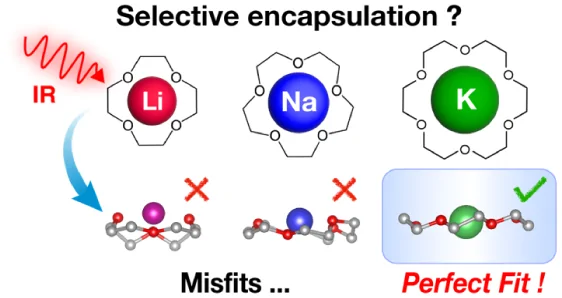

## Introduction

Macrocyclic molecules, both naturally occurring and synthetic, have been shown to exhibit size-selectivity to a variety of singly- and multiply charged ions.^1–9^ Among these species, the crown ethers have a long history and arguably marked the beginning of supramolecular chemistry.^10–13^ 12-Crown-4 (12C4), 15-Crown-5 (15C5), and 18-Crown-6 (18C6) exhibit various degrees of binding selectivity for specific cations in solution despite their simple cavity structure, by orienting electronegative oxygens toward the metal ion. While this structural flexibility is shared by many other ionophores,^1–9^ *vide infra*, the high degree of uniformity and structurally similar formulation (−(CH_2_)_2_O−)_*n*_, is unique, and amenable to conformational verification, making them excellent model systems for investigating the relationship between ion selectivity and molecular conformation. The capability of crown ethers has opened the door to several broad and fruitful areas, including synthetic, analytical, and material science, as well as a wide range of applications across other fields.^14–18^ From an application perspective, the growing demand for Li^+^ extraction from sustainable resources such as brines, ores, and seawater has recently made ion extraction exploiting the selectivity of crown ethers an intensive research topic.^19–23^ As a result, more than 50 years after their discovery, crown ethers and their derivatives continue to play a central role in a broad field of chemistry, with new molecular systems being developed one after another.

Nevertheless, the molecular origin of their selective behavior is not fully understood, making its mechanism an important challenge in the field of physical chemistry. The simplest model explaining ion selectivity of crown ethers is the “matching” concept, in which a crown ether is expected to exhibit high affinity for a metal ion whose ionic radius closely matches the nominal 2-D cavity size of the macrocycle (e.g., 12C4 for Li^+^, 15C5 for Na^+^, and 18C6 for K^+^ as seen in Figure 1a).^24,25^ Interestingly, these 2-D depictions continue to persist,^26^ despite the ability for these inherently flexible molecules to adopt a wide range of 3-D conformations in response to ion binding, which alters the effective size of the cavity. The concept of ionophore structural flexibility is well-known as can be seen by numerous examples in the literature, such as valinomycin^27,28^ (selective sequestering of K^+^), beauvericin^29,30^ (selective binding of biologically relevant mono- and dications) and macropa-derivatives^31,32^ (size-specificity of alkaline and rare-earth di- and trications), but correlating this structural flexibility with ion-selectivity at the molecular level requires a complete analysis of the ion–ionophore complex, utilizing both theoretical and spectroscopic methods. The alkali crown ether system is ideally suited for such an investigation.

**Figure 1:**
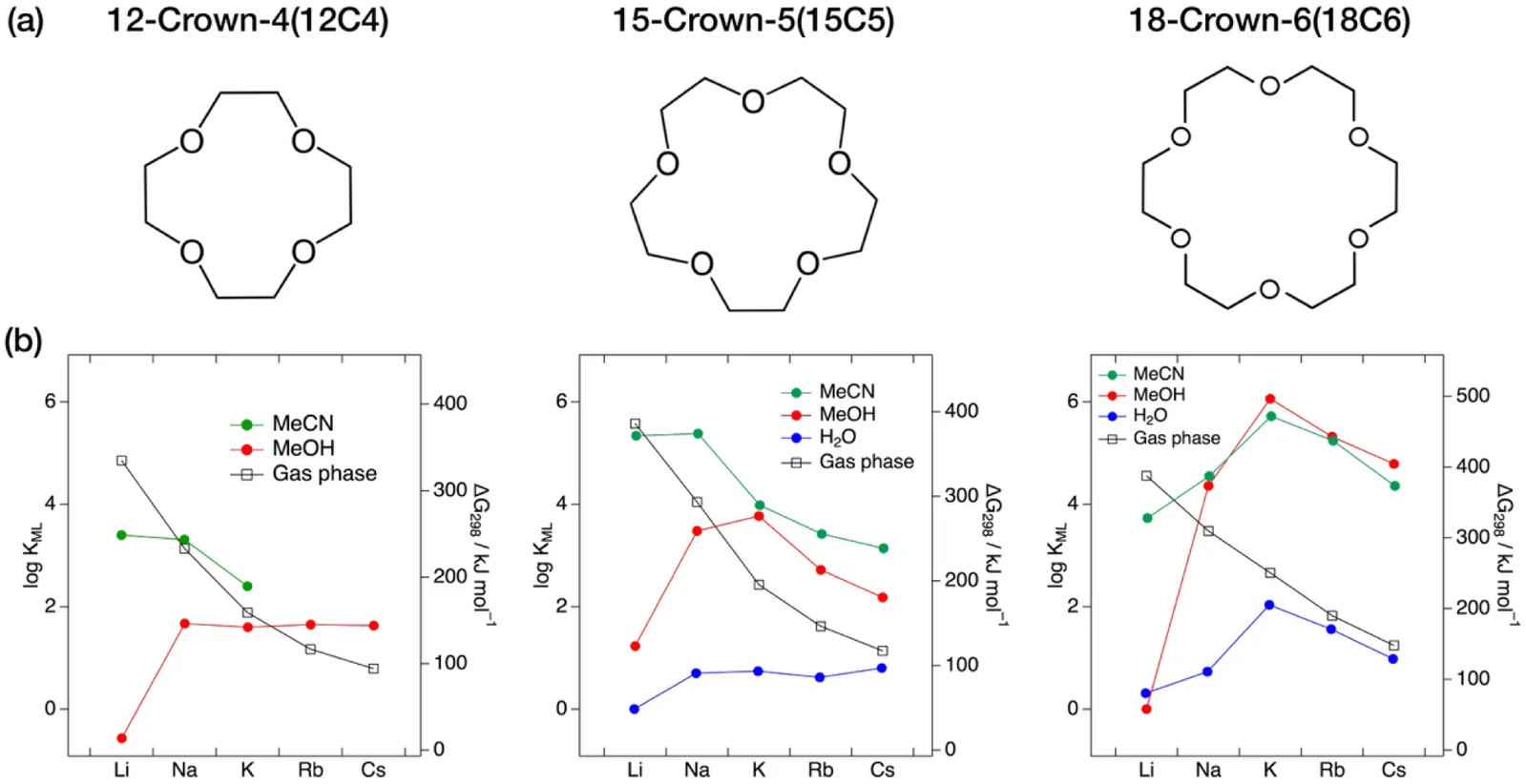
(a) Chemical structures of 12-crown-4 (12C4), 15-crown-5 (15C5), and 18-crown-6 (18C6). (b) Solution-phase equilibrium constants (circles)^33–41^ and gas-phase binding energies (squares) with alkali metal ions calculated at the B3LYP/6-311++G (d,p) level. The binding energies correspond to the structures assigned later (Figure 2).

Figure 1b summarizes the binding constants of 12C4, 15C5, and 18C6 in various solvents, as well as their gas-phase binding energies in the absence of solvent effects.^33–41^ Note that the intrinsic gas-phase binding energies of alkali metal ions to crown ethers decrease monotonically with increasing ionic radius for all three systems, indicating that, the binding affinity is dominated by ionic charge density rather than size complementarity. While the equilibrium constants associated with 18C6 exhibit a consistent trend, showing a maximum affinity for K^+^ across different solvents, neither 12C4 nor 15C5 exhibit clear Li^+^ or Na^+^ selectivity in solution. Additionally, the equilibrium constants can vary over four to five orders of magnitude for specific ion-crown ether systems depending on the solvent. These results highlight the inherent limitations of a simple matching model, suggesting that intrinsic binding of the ions by the crown ethers, the dominant noncovalent interaction, heavily influences how the different solvents compete for the ions. Any investigation of solvent interactions, as a first step, requires a systematic characterization of the intrinsic gas-phase structures at the molecular level before ion preference and selectivity can be modeled in solution. Such structural insight is essential for revealing the underlying host–guest interactions and for establishing guidelines for rational, structure-based molecular design.

In practice, direct access to binding structures in solution is nontrivial, as multiple competing noncovalent interactions such as metal–crown ether, metal–solvent, solvent–crown ether, and solvent–solvent interactions are intricately intertwined, making it difficult to disentangle their individual contributions based solely on condensed-phase data.^42–47^ As noted above, understanding the intrinsic ion-crown ether interaction is the essential starting point, and gas-phase studies provide a valuable approach to isolate and characterize these interactions, which can then be followed by stepwise solvation. For this reason, metal ion–crown ether complexes and their microsolvated clusters have been extensively studied using mass spectrometry, vibrational spectroscopy, and theoretical methods to elucidate the detailed interactions underlying ion selectivity.^48–66^

Gas-phase vibrational spectroscopy has proven to be a powerful tool for investigating molecular structures and dynamics at the microscopic level. Recent advances in cryogenic spectroscopy combining electrospray ionization (ESI) with cryogenic ion trap techniques have enabled high-resolution spectral measurements, allowing for detailed structural characterization of a wide range of molecular systems.^67–72^ While an extensive body of work exists on metal ion–crown ether complexes and their solvated clusters, extracting binding structures directly from experimental spectra has proven nontrivial. This difficulty arises from the intrinsically complex vibrational signatures of crown ethers, which complicate theoretical interpretation and obscure structural information on the crown ether backbone. Consequently, despite the accumulation of experimental spectra, quantitative agreement with theory has been limited, and the unambiguous *sequentially solvated* structures of many encapsulated species remain unresolved.

Recently, we have found a strategy to overcome the challenges described above, i.e., cryogenic IR spectroscopy combined with anharmonic vibrational analysis to investigate the CH stretch region of M^+^ 18C6­(H_2_O)*_n_* clusters.^72^ This spectrally rich region (with 12 CH_2_ groups as seen in Figure 1) is strongly influenced by anharmonic coupling between CH stretch fundamentals and CH_2_ bending overtones yielding highly complex band patterns. Nevertheless, by combining cryogenic spectroscopy with a local mode model developed by the Sibert and Zwier groups,^73–78^ we resolved and assigned the bands to specific structures of M^+^ 18C6­(H_2_O)_*n*_. This study revealed that CH stretch spectra are highly sensitive to the conformational flexibility, demonstrating how water molecules disrupt the strong binding of Li^+^ and Na^+^ to 18C6, and can serve as powerful fingerprints for probing variations in these encapsulated complexes. This integrated experimental–theoretical approach thus offers a direct route to elucidating the structures of the ion-crown ether complexes, addressing a long-standing challenge to advance the field.

Here, we would like to better address the general concept of ion selectivity by crown ethers based on the above strategy. The first step is to systematically apply the CH structural analysis to alkali metal ion–crown ether complexes with three different ring sizes, 12C4, 15C5, and 18C6, with Li^+^, Na^+^, K^+^, Rb^+^ and Cs^+^. Despite their widespread use across diverse fields, detailed structural studies, particularly for 12C4 and 15C5, have remained relatively limited. While 12C4 and related 14C4 ethers have been attracting the avid attention of researchers in terms of Li^+^ extraction and recycling,^19–23^ no clear explanation on Li^+^ selectivity has been rationalized as we described in Figure 1. Using a unified experimental and computational framework, we carried out a systematic study, enabling us to establish the intrinsic gas-phase structures and to elucidate how crown ethers optimize cavity geometries to different metal ions. Characterizing these intrinsic structures before probing solvent interactions is essential for understanding how solvation then perturbs binding geometries and ultimately dictates ion selectivity in solution. Importantly, our results refine the conventional matching concept where crown ethers can adopt a wide range of conformations. In addition, the sharp solvent-dependence on the equilibrium constants for Li^+^ and Na^+^ with 12C4 and 15C5, points out the importance of solvent competition, with potential application for selective extraction. Overall, our findings provide a definitive molecular-level picture for the further investigation of solvent effects and ion selectivity in solution and the rational design of ion-selective molecules.

## Experimental Section

Infrared predissociation (IRPD) spectra of M^+^12C4, M^+^15C5, and M^+^18C6 (M = Li, Na, K, Rb, and Cs) complexes were measured using a cryogenic double ion trap setup (Figure S1).^72^ The alkali metal ion crown ether complexes were generated by electrospray ionization from methanol solutions of alkali metal chloride MCl and crown ether (for concentrations, ∼10^–5^ M, respectively). The generated ions are introduced into the vacuum via an ion funnel and then selected by a quadrupole mass spectrometer and transported into a cryogenic 3-dimensional quadrupole ion trap (QIT) kept at ∼ 4 K by a closed-cycle two-stage He refrigerator. As ions were injected to the QIT, a mixture of hydrogen (20%) and helium (80%) buffer gas was introduced to the QIT via a pulsed valve. Colliding with the buffer gas molecules, the ions were trapped and cooled down to ∼10 K (vibrational temperature), allowing the formation of weakly bound H_2_-tagged adducts. The trapped ions were interrogated with a pulsed, tunable OPO/OPA IR laser (LaserVision). Resonant absorption of a single IR photon was sufficient to dissociate the weakly bound H_2_ tag molecules. The resulting photofragment ions were ejected and detected by a time-of-flight mass spectrometer (TOF-MS). Infrared predissociation (IRPD) spectra were measured by monitoring the yield of the photofragment ions as a function of the wavenumber of the IR laser.

## Computational Details

Conformational searches were performed using Grimme’s CREST program introduced by Pracht et al.^79^ The generated structures were then subjected to geometry optimization using DFT as implemented in Gaussian16.^80^

DFT calculations were performed for the M^+^12C4, M^+^15C5, and M^+^18C6 complexes. Geometry optimizations were carried out using the B3LYP functional and lanl2dz (Rb, Cs)/6-311++G­(d,p) (Li, Na, K) basis sets. The relative zero-point corrected energies and Gibbs free energies at 120/298 K of the optimized structures were compared to identify the lowest-energy conformer for each system (Figures S2–S16).

To calculate the CH stretch spectra of alkali metal ion–crown ether complexes, we employed the local-mode anharmonic model, compatible with the computational level noted above, developed by the Sibert group.^73–78^ The modeling of the CH stretch region followed the same protocol as that used in our previous study on gas-phase M^+^ 18C6­(H_2_O)_*n*_ complexes.^72^ In this model, a local mode Hamiltonian was constructed to describe states with one quantum of CH stretch excitation coupled to nearly degenerate overtones and combination states of CH_2_ bending vibrations. Both harmonic and anharmonic terms are included in the Hamiltonian matrix. The harmonic term was derived from normal modes calculations performed at the B3LYP/6-311++G­(d,p) level of theory. Normal modes associated with CH stretch, CH_2_ scissor, and wag vibrations were localized via an orthogonal transformation. Dipole derivatives were similarly transformed into the local-mode representation. The model Hamiltonian includes CH stretch fundamentals as well as overtones and combination bands of CH_2_ scissor and wag modes. The diagonal elements of the Hamiltonian were scaled by factors of 0.961 for CH stretch, 0.975 for CH_2_ scissor, and 0.980 for CH_2_ wag modes. The Fermi coupling between the CH stretch and CH_2_ bending modes was treated following the procedures described in recent work.^7,78^ The Hamiltonian matrix was diagonalized to obtain transition frequencies and transition dipole moments. The calculated anharmonic spectra were convoluted with a Lorentzian function with a full width at half-maximum (FWHM) of 5 cm^–1^ for comparison with the experimental spectra. Binding energies for the assigned structures were calculated at the same levels of theory. The numerical values are listed in Table S1.

## Results and Discussion

Figure 2 presents the IRPD spectra of M^+^ 12C4, M^+^ 15C5, and M^+^ 18C6 (M = Li^+^ – Cs^+^) in the CH stretch region. Distinct spectral patterns are observed as a function of both crown ether ring size and metal ion size, indicating pronounced structural sensitivity of the CH stretch vibrations. The IR spectra of M^+^ 15C5 complexes are reported here for the first time. In addition, experimental advances for cryogenic measurements yield substantially better-resolved features compared to previous studies^55^ on M^+^ 12C4 and M^+^ 18C6, enabling detailed structural analysis. As the size of the crown ether increases, the density and breadth of the CH bands increase for each alkali metal ion, reflecting both the growth in the number of CH groups and anharmonic interactions.

**Figure 2:**
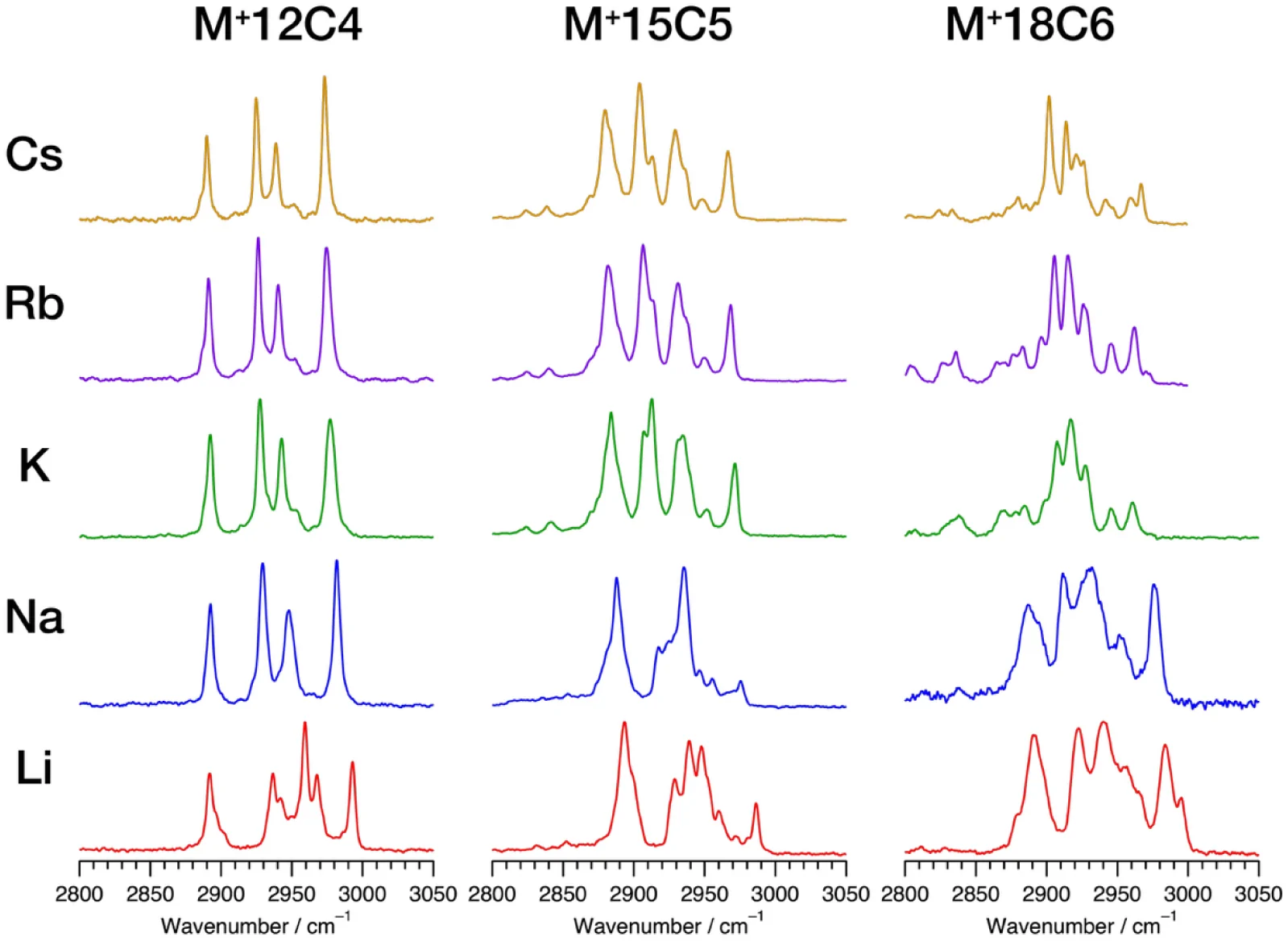
IRPD spectra of M^+^12C4, M^+^15C5, and M^+^18C6 (M = Li, Na, K, Rb, and Cs) complexes in the CH stretch region. The spectra of M^+^18C6 are taken from ref^72^.

For M^+^12C4, the spectra are nearly identical for all ions, except Li, consisting mainly of four characteristic bands. In contrast, the spectrum of Li^+^ exhibits more strongly split features compared to other alkali metal ion complexes. In particular, the band near ∼2960 cm^–1^ shows enhanced intensity for Li^+^12C4, whereas for the larger ions the dominant feature is observed around ∼2930 cm^–1^. These differences indicate that the binding structure for Li^+^ is distinct from those adopted by the Na–Cs complexes.

For M^+^15C5, the IR spectra exhibit different trends from those observed for M^+^12C4. The spectra of Li^+^ and Na^+^ show subtle yet clear differences in their band patterns: Li^+^15C5 displays multiple closely spaced peaks around ∼2940 cm^–1^, whereas the corresponding feature for Na^+^15C5 collapses into an almost single dominant band. In contrast, for the {K^+^–Cs^+^} group, a new band appears near ∼2910 cm^–1^, and the overall profiles for this group are highly similar, clearly distinguishing them from the Li^+^ and Na^+^ complexes. These spectral trends for Li^+^, Na^+^ and {K^+^–Cs^+^} bound to 15C5, reflect a pronounced structural evolution with increasing ionic radii and indicate three different binding motifs. In contrast to 12C4 and 18C6, the odd number of oxygens in 15C5 may prevent symmetric binding pattern, such as *C*_4_ or C_3_ and promote structures with nonplanar grouping of the oxygen atoms.

For M^+^18C6, the IR spectra exhibit significantly more complex band profiles than those observed for 12C4 and 15C5, with broader band contours across the CH stretch region. The spectra of Li^+^ and Na^+^ show closely similar overall patterns, as do those of K^+^ through Cs^+^. A marked difference is observed in the distribution of spectral intensity; for Li^+^ and Na^+^, the IR intensity is broadly dispersed over the 2870–3000 cm^–1^ region, whereas for the heavier ions (K^+^–Cs^+^), the intensity is concentrated predominantly between 2900 and 2950 cm^–1^. In our previous work, the distinct transition between the smaller ions and K^+^ was unambiguously assigned to buckling of the 18C6 ring (Figure 3) by the smaller ions, which perturbs the local CH environments and leads to a drastic change in the spectral features. The observation that the spectra of Rb^+^ and Cs^+^ closely resemble that of K^+^ therefore indicates that these larger ions adopt similar binding structures. This binary classification into Li^+^/Na^+^ and K^+^–Cs^+^ mirrors the behavior observed for 15C5, an odd-membered crown ether, rather the even-membered 12C4. Apparently, the binding geometries to Li^+^ and Na^+^ strongly depend on the crown-ether ring size, whereas K^+^–Cs^+^ adopt essentially the similar binding motif representative to each crown ether. Can these spectral observations be used to develop a fundamental insight into the binding geometries and differences between these 15 ion-crown ether complexes?

**Figure 3:**
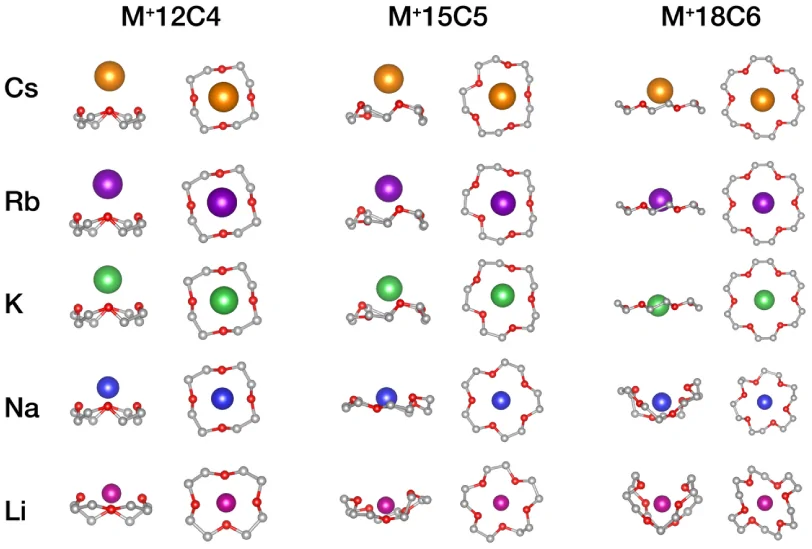
Computed minimum energy structures for M^+^12C4, M^+^15C5, and M^+^18C6 (M = Li, Na, K, Rb, and Cs) complexes at the B3LYP/6–311++G­(d,p) (left: side view; right: top view). M^+^18C6 structures are based on data from ref^72^.

To understand whether the spectroscopic trends described above can be explained or rationalized by theory, we calculated the lowest-energy structures of M^+^ 12C4, M^+^15C5, and M^+^18C6 complexes (B3LYP/6-311++G­(d,p)) as presented in Figure 3. Additional low-energy structures for conformers of all complexes are provided in the Supporting Information (Figures S2–S16). As crown ethers are highly flexible molecules exhibiting complex conformational landscapes, these are constrained by both the macrocyclic framework and the preferred coordination environment of the bound ion. As a result, numerous low-lying conformers were found, which differed primarily in the dihedral angles along the −O–CH_2_–CH_2_–O– linkages.

For M^+^12C4, two distinct binding motifs were found among the lowest-energy conformers, with Li^+^ exhibiting a unique binding pattern. Surprisingly, even for Li^+^, the 12C4 cavity was too small to fully encapsulate the ion which resided above the plane of the ether. While the Na^+^–Cs^+^ complexes exhibited *C*_4_ symmetry (see top view), the Li^+^ complex adopted a *C*_*s*_ symmetry (a *C*_4_ structure was found within 1 kJ/mol; see Figure S2). No *C*_*s*_ binding motif, characteristic of Li^+^, was observed in the low-energy conformers of the Na^+^–Cs^+^ complexes. Figure 4 compares the experimental IR spectra with the anharmonic spectra calculated for the structures shown in Figure 3 using the local mode anharmonic model for Li^+^, Na^+^ and K^+^ (detailed comparisons for other low-energy conformers and for Rb^+^ and Cs^+^ are included in the Supporting Information, Figures S2–S31). The theoretical spectra show excellent agreement with the experimental IR spectra, reproducing not only the overall band profiles but also the band positions and their systematic ion-dependent frequency shifts. This excellent agreement allows us to confidently assign the observed IR spectra to the corresponding minimum-energy structures.

**Figure 4:**
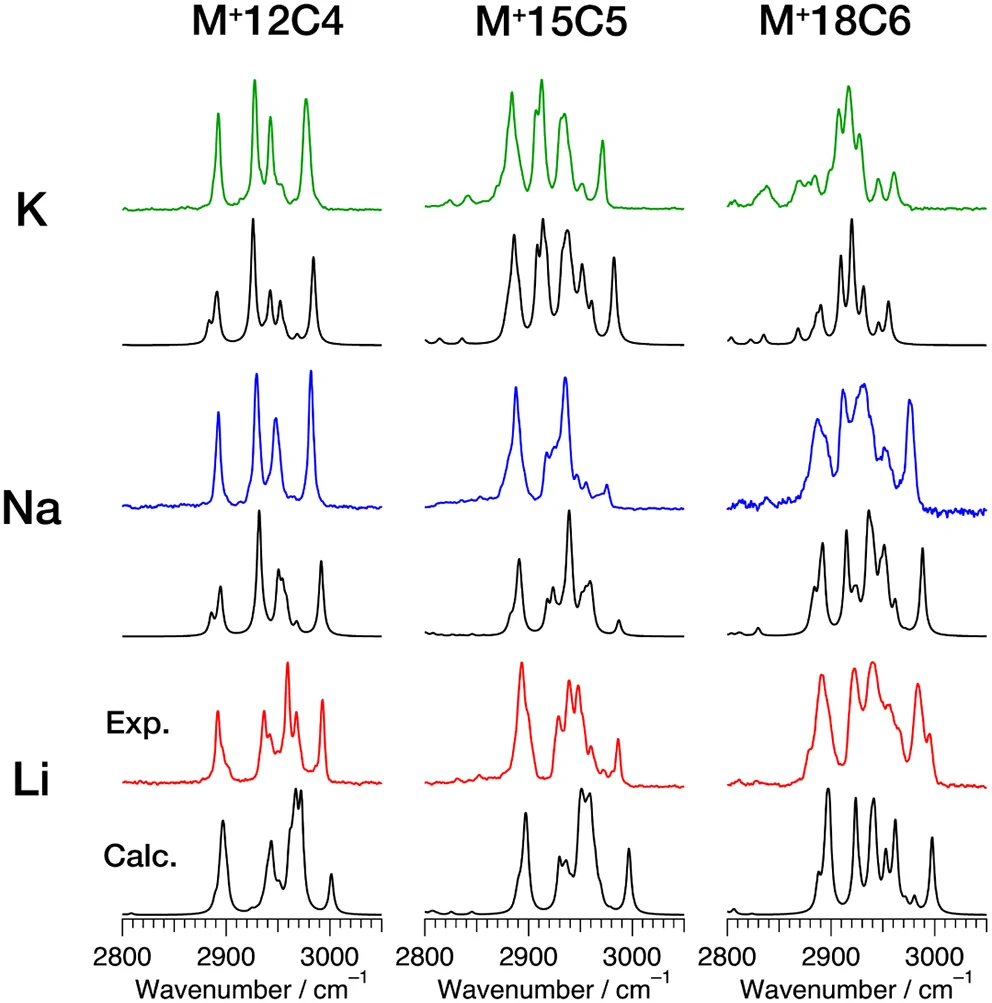
Comparison of experimental and calculated (black) IR spectra of M^+^12C4 (M = Li (red), Na (blue), and K (green)), M^+^15C5, and M^+^18C6 complexes at the B3LYP/6-311++G­(d,p) level in the CH stretch region. Data for 18C6 were taken from ref^72^.

Having established the correspondence between structure and IR spectral features for 12C4, we now turn to the 15C5 complexes, where the odd-membered ring introduces additional structural flexibility. The Li^+^ complex adopts a strongly distorted conformation in which the crown ring wraps around the small ion, leading to partial encapsulation. In contrast, the Na^+^ complex exhibits a less distorted structure, with the crown ether adopting a comparatively flatter conformation.

For K^+^ through Cs^+^, a binding motif distinct from that of Na^+^ is observed. In these complexes, the ions are clearly positioned above the crown ring rather than within the cavity, and the overall structural motifs are highly similar across this ion series. Although Na^+^-type structures can also be identified for the K^+^–Cs^+^ complexes, they lie at significantly higher energies, ∼5 kJ mol^–1^ for K^+^ and ∼10 kJ mol^–1^ for Rb^+^ and Cs^+^. These results indicate that the lowest-energy binding structures of M^+^ 15C5 can be classified into three categories, consistent with the experimental trends. Notably, the anharmonic IR spectra calculated for these structures show excellent agreement with the experimental spectra, validating the calculated structures.

For the 18C6 complexes, two distinct binding motifs were identified among the lowest-energy structures from our previous work.^72^ Li^+^ and Na^+^ adopt buckled conformations, whereas K^+^ through Cs^+^ form flat crown ether structures with 3-fold symmetry (Figure 3). Li^+^ and Na^+^ are too small to stabilize a flat 18C6 conformation, while K^+^–Cs^+^ stabilize the flat crown ring. The K^+^ complex exhibits high *D*_*3* *d*_ symmetry, reflecting the central position of the ion within the cavity. In contrast, for the larger Rb^+^ and Cs^+^ ions, the symmetry of the complexes is reduced to *C*_*3v*_, as the ions are displaced above the ring. In our previous work, the Li^+^, Na^+^, and K^+^ complexes were unambiguously assigned to their respective lowest-energy conformers based on direct comparison between experimental and theoretical IR spectra.

Taken together, the combined experimental and theoretical analysis establish a consistent and unambiguous structural assignment for M^+^–crown ether complexes from 12C4 to 18C6 by exploiting the CH stretch region as a fingerprint for the first time. To compare the ion–complex structures of M^+^12C4, M^+^15C5, and M^+^18C6 in greater detail, we utilize a set of structural parameters from the assigned lowest-energy conformers to quantify these differences as shown in Figure 5a. Here, R_O_ denotes the distance between the metal ion and the oxygen atoms of the crown ether. Parameters h_O_ and h_M_ represent the distances of the oxygen atoms and the metal ion, respectively, from the mean plane defined by all oxygen atoms of the crown ether. These geometric parameters provide both insight and a quantitative measure of ion accommodation (i.e., the degree of fit within the crown ether cavity), crown ring distortion, and deviations from planarity across different crown ether sizes and metal ions. This geometric analysis has previously been applied to metal ion–crown ether complexes by the Inokuchi group.^58^ Variations in these structural parameters for the conformers assigned in this work are shown in Figure 5b–j.

**Figure 5:**
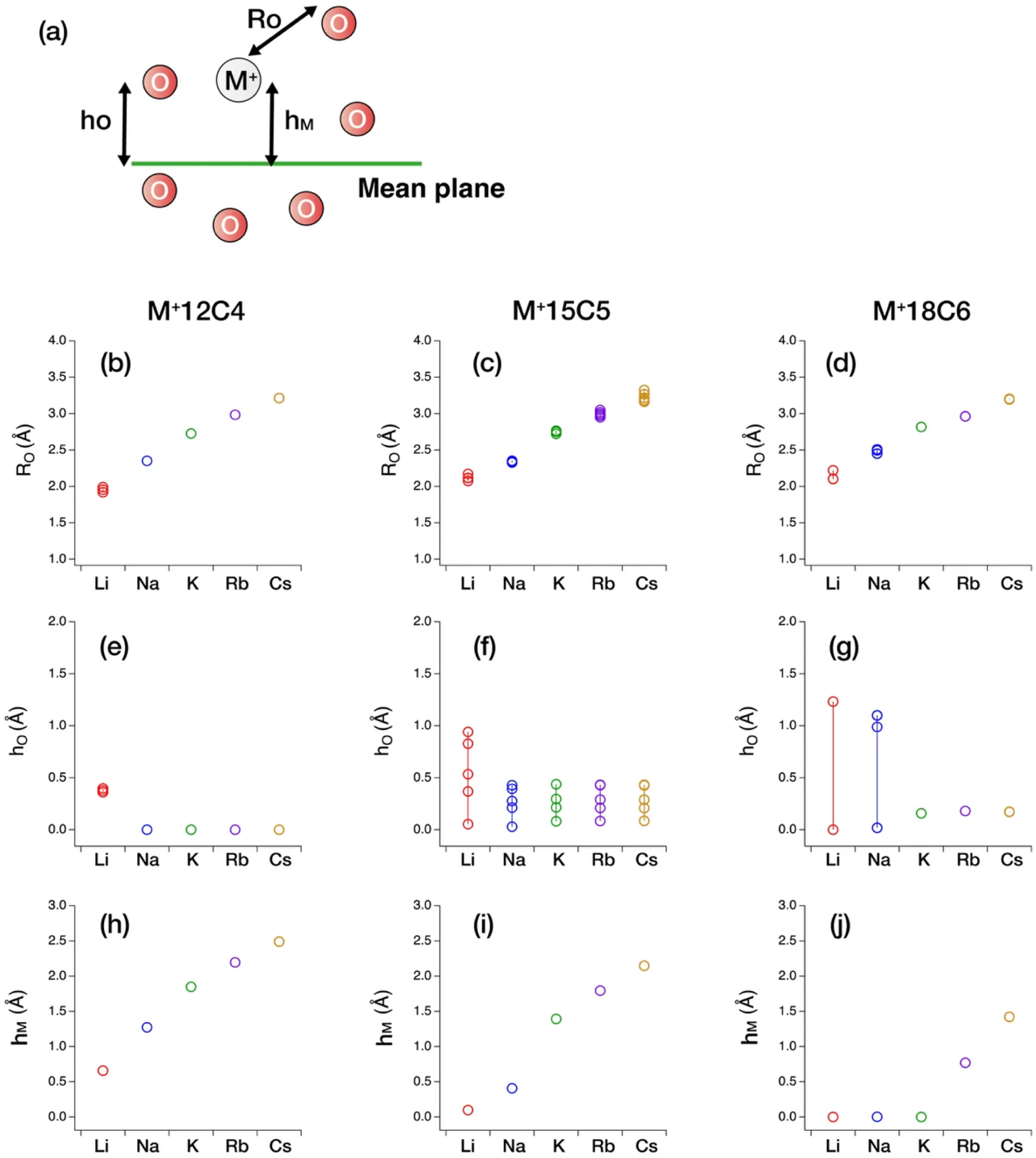
Computed geometrical parameters for M^+^12C4 (M = Li, Na, K, Rb, Cs), M^+^15C5, and M^+^18C6 complexes at the B3LYP/6-311++G -(d,p) (Figure 3) to quantify the binding structures of crown ethers. (a) Definition of geometric parameters. (b–d) Ion-etheric oxygen distances. (e–g) Distances of the etheric oxygen atoms from the mean plane of all the oxygen atoms. (h-j) Distances of the alkali metal ion from the mean plane of all the oxygen atoms.

For the M^+^ 12C4 complexes, as seen in Figure 5b and 5e, and Table 1, the structure of the Li^+^ species is unique in comparison to the other ions. The oxygen atoms do not form a plane (h_O_ is ∼0.4 Å) and there is a spread in the values of R_O_ = 1.96 (3) Å. This reflects a three-dimensional arrangement of the oxygen atoms, in which two oxygen atoms are located above and two below the mean plane of the crown ring as can be seen in Figure 3. In contrast, for the Na^+^–Cs^+^ complexes, h_O_ approaches zero with coplanar oxygen atoms, and R_O_ has a uniform value for each ion. This uniformity is clearly displayed in the spectra (Figure 2) and calculated structures (Figure 3) with the *C*_4_ symmetry preserved as each larger ion recedes from the crown ether as reflected in the increasing values of R_O_ and h_M_. As h_M_ is nonzero for all ions and increases monotonically from Li^+^ to Cs^+^, this demonstrates that even the smallest ion, Li^+^, is too large to be fully accommodated within the 12C4 cavity. This result contrasts with the conventional description that Li^+^ is well matched to the 12C4 cavity.

**Table 1:** Comparison Between Values of Average R_O_ for the M^+^-Crown Ether Complexes from the Calculations and the Sum of the M^+^ Ionic Radii^81^ (Coordination Number = 6) and Oxygen van der Waals Radius (1.52 Å).^82^ ^a^

Ion	Li	Na	K	Rb	Cs
R_M_^+^ + R_OvdW_ (Å)	2.28	2.54	2.90	3.04	3.19
R_O_(M^+^12C4) (Å)	1.96(3)	2.35(0)	2.73(0)	2.98(0)	3.21(0)
R_O_(M^+^15C5) (Å)	2.12(3)	2.34(1)	2.75(2)	3.00(4)	3.23(6)
R_O_(M^+^18C6) (Å)	2.18(6)	2.48(2)	2.82(0)	2.96(0)	3.20(0)

a Values in parentheses represent the standard deviation.

For the M^+^15C5 complexes, it appears that the odd number of oxygen atoms, prevents any of the ions from being equidistant to each atom (R_O_) as seen in Table 1. As a result, the ions exhibit relatively large h_O_ values (Figure 5f) with broader distributions, a significant contrast in comparison to 12C4. Since the spread in h_O_ directly reflects the degree of crown ring buckling, this result demonstrates that Li^+^ induces the strongest distortion. For 15C5, h_M_ does not display a monotonic increase with ion size, but instead has a sharp discontinuity between Na and K (Figure 5i). This underscores the near, yet incomplete encapsulation of Li^+^ and Na^+^ by 15C5. While Li^+^ is located near the center of the cavity (h_M_ ≈ 0.1 Å), it is not quite a perfect fit due to the buckled 15C5 ring. Na^+^ exhibits a slightly larger displacement (h_M_ ≈ 0.4 Å), remaining close to the crown ether cavity but again indicating encapsulation is not quite complete. The odd-numbered 15C5 is structurally disfavored in achieving a uniform sequestration of these two smaller ions. The large jump in h_M_, going from Na^+^ to K^+^, makes clear that K^+^ (as well as Rb^+^ and Cs^+^) binds on top of 15C5. As seen in Table 1, Figures 2 and 3, K^+^ – Cs^+^ are stabilized at positions displaced above the ring with uniform configurations and IR spectra.

With 18C6, Li^+^ and Na^+^ exhibit the largest range in h_O_ values (Figure 5g) compared to those observed for 12C4 and 15C5. This behavior reflects the highly three-dimensional nature of the cavity, in which the oxygen atoms strive to coordinate the metal ion in an approximately octahedral geometry. In contrast, for K^+^–Cs^+^, h_O_ is nearly zero, indicating the formation of an almost planar structure formed by the oxygen atoms. These results highlight the high conformational flexibility of 18C6, which allows the oxygens to bind metal ions of different sizes by switching between three-dimensional and planar binding motifs. For the M^+^18C6 complexes, Li^+^ through K^+^ are encapsulated within the crown ether cavity (h_M_ ≈ 0). For Li^+^ and Na^+^ this is accomplished by a 3-D structure adopted by the oxygen atoms to maximize the interaction with the ions. K^+^, in contrast, is essentially a perfect fit to the planar cavity, allowing the ion to sit in the plane formed by the six oxygen atoms with a uniform R_O_ = 2.82 Å and a minimum value of h_O_ = 0.16 Å. Taken in combination, this demonstrates that K^+^ is a perfect match to 18C6, which we define to be selective encapsulation. While the larger Rb^+^ and Cs^+^ ions are too large to be accommodated by 18C6, with values of h_M_ of ∼0.8 and 1.4 Å (Figure 5j) respectively above the crown ring, the structure of the ether backbone is unchanged from that of K^+^18C6, based on the IR spectra (Figure 2) and calculated structures (Figure 3).

Crown ether cavities are highly flexible and responsive to different ionic radii, capable of adjusting the crown cavity to maximize metal ion–crown ether interactions. These variations in ion size induce pronounced structural rearrangements, by allowing the etheric oxygen atoms to coordinate the metal ion within the limits of the covalent bond structure of the ring. This picture is fundamentally incompatible with the conventional size-matching model, which assumes a static crown ether cavity and does not account for flexibility. These results indicate, with regards to optimal host–guest matching, 12C4 does not fully accommodate any alkali metal ions. Even Li^+^ is too large to be encapsulated, with h_M_ > 0.5 Å and h_O_ ≠ 0. While 15C5 may appear to be optimal for Li^+^ with h_M_ ∼0.1 Å, it exhibits the widest spread in h_O_, ∼0–1 Å, twice that of the other ions, indicating nonuniform binding to 15C5. With respect to selective encapsulation, only 18C6 can accomplish this feat with K^+^, as h_M_ = 0 Å and h_O_ = 0.16 Å for all six oxygens. Note in Figure 1b, only 18C6 displays a uniform preference for K^+^ in solution for the three standard solvents: H_2_O, CH_3_OH and CH_3_CN, while 15C5 and 12C4 favor different ions, depending on the solvent. This may indeed reflect the selective encapsulation of K^+^ by 18C6, where the structure of the ion complex was shown to be unaffected by hydration with up to three water molecules.^72^

## Conclusion

The present study combines cryogenic IR spectroscopy with anharmonic analysis in the CH stretch region to determine the detailed gas-phase structures of a series of alkali metal ion–crown ether complexes with different cavity sizes. By comparing the experimental spectra with theoretically predicted CH stretch spectra, we unambiguously assign the conformations of each M^+^–crown ether complex. Analysis of the determined geometries reveals that, in the gas phase, no alkali metal ion is optimally accommodated by 12C4 or 15C5. While Li^+^–K^+^ are encapsulated by 18C6, only K^+^18C6 exhibits selective encapsulation. Detailed analysis of the molecular-level structures identifies deficiencies in the conventional size-matching model, which assumes a simple relationship between ionic size and planar cavity hole size. The molecular flexibility of the crown ethers gives rise to diverse encapsulation motifs for alkali metal ions. For 12C4 and 15C5, no alkali metal ion can adopt a configuration with the ion positioned in the plane of the oxygen atoms (h_M_ = 0 Å) and with the oxygen atoms uniformly positioned in that plane (h_O_ = 0.16 Å). With respect to 15C5, the odd number of oxygen atoms and limited flexibility for the ring, preclude the ions from the degree of encapsulation observed for 18C6. This could explain why different solvents favor different ions in terms of solution-phase association constants. Such behavior could be exploited for separations of specific ions by allowing binding to an ionophore-bound column with one solvent and then stripped by a different solvent.

Although the guest metal ions are essentially spherical objects, the host crown ethers undergo substantial conformational rearrangements by adapting the shape of the etheric ring to maximize host–guest interactions. This behavior refines the traditional static size-matching picture and highlights host flexibility as a key factor governing the structural stability of crown ether complexes. Moreover, the well-defined gas-phase structures obtained in this study provide an excellent platform for investigating solvent interactions with these complexes. As shown by the experimental IR spectra and computational results, the CH stretch region is an accurate “fingerprint” of the crown ether configuration as it binds to the ion. When the IR spectra for a series of ions is unchanged (Na^+^ to Cs^+^ for 12C4, K^+^ to Cs^+^ for 15C5 and 18C6), the structure of the crown ether ring is independent of the position of the ion. For Li^+^ and Na^+^ (except 12C4), strong electrostatic interactions with the crown ether led to a pronounced buckling and 3-D structure of the ion–ionophore species, as these systems seek to maximize the ion-oxygen interactions. Since ion selectivity in solution is governed by a balance between metal ion–crown ether interactions and solvation effects, systematic investigations are essential to reveal sequestration of specific ions. These include sequential solvation of metal ion-crown ether complexes, dependence on solvent type and number of solvent molecules, to bridge the gap between gas-phase and condensed-phase environments.^61–63,72^ Such efforts, which are currently underway, are essential for modeling ion selectivity of crown ethers and for elucidating solvation effects in ion recognition. More broadly, because CH stretch vibrations are ubiquitous in organic molecules, the experimental and computational strategy demonstrated here provides a general framework for probing the molecular structure in a wide range of ionophores.

## Supplementary Material



## Notes

Description of the experimental setup, binding energies of M^+^12C4, M^+^15C5, and M^+^18C6 (M = Li, Na, K, Rb, and Cs), low-energy conformers of M^+^12C4, M^+^15C5, and M^+^18C6 (M = Li, Na, K, Rb, and Cs), comparison between experimental and calculated spectra for M^+^12C4, M^+^15C5, and M^+^18C6 (M = Li, Na, K, Rb, and Cs), and xyz format for M^+^12C4, M^+^15C5, and M^+^18C6 (M = Li, Na, K, Rb, and Cs) (PDF)
