# Supplementary material for: Spectroscopic Tracking of Size Selectivity in Crown Ethers: A Unified Molecular Picture of Encapsulation
Source: J Am Chem Soc. 2026 Aug 5;148(32):34209–18. doi: 10.1021/jacs.6c05693 (PMC13495753; doi:10.1021/jacs.6c05693)
Supplement: Supplementary file 1 [file ja6c05693_si_001.pdf]

Supporting Information for

Spectroscopic Tracking of Size Selectivity in Crown Ethers: A Unified Molecular Picture of Encapsulation

Ryu Sakuma,[‡] *Keisuke Hirata,*[‡] *James M. Lisy,**[⊤⊥] *Masaaki Fujii**[†ǀ⊤] *and Shun-ichi Ishiuchi**[‡]

[‡]Department of Chemistry, School of Science, Institute of Science Tokyo, 2-12-1 Ookayama, Meguro-ku, Tokyo 152-8550, Japan

[†]Laboratory for Chemistry and Life Science, Institute of Integrative Research, Institute of Science Tokyo, 4259 Nagatsuta-cho, Midori-ku, Yokohama 226-8503, Japan

[ǀ]Research and Development Initiative, Chuo University, 1-13-27 Kasuga, Bunkyo-ku, Tokyo 112-8551, Japan

[⊤] Institute of Integrative Research (IIR), Institute of Science Tokyo, Nagatsuta-cho, Midori-ku, Yokohama 226-8503, Japan

[⊥]Department of Chemistry, University of Illinois at Urbana-Champaign, Urbana, IL 61801, USA

* Corresponding authors

JML: j-lisy@illinois.edu, SI: ishiuchi.s.aa@m.titech.ac.jp, MF: mfujii@res.titech.ac.jp

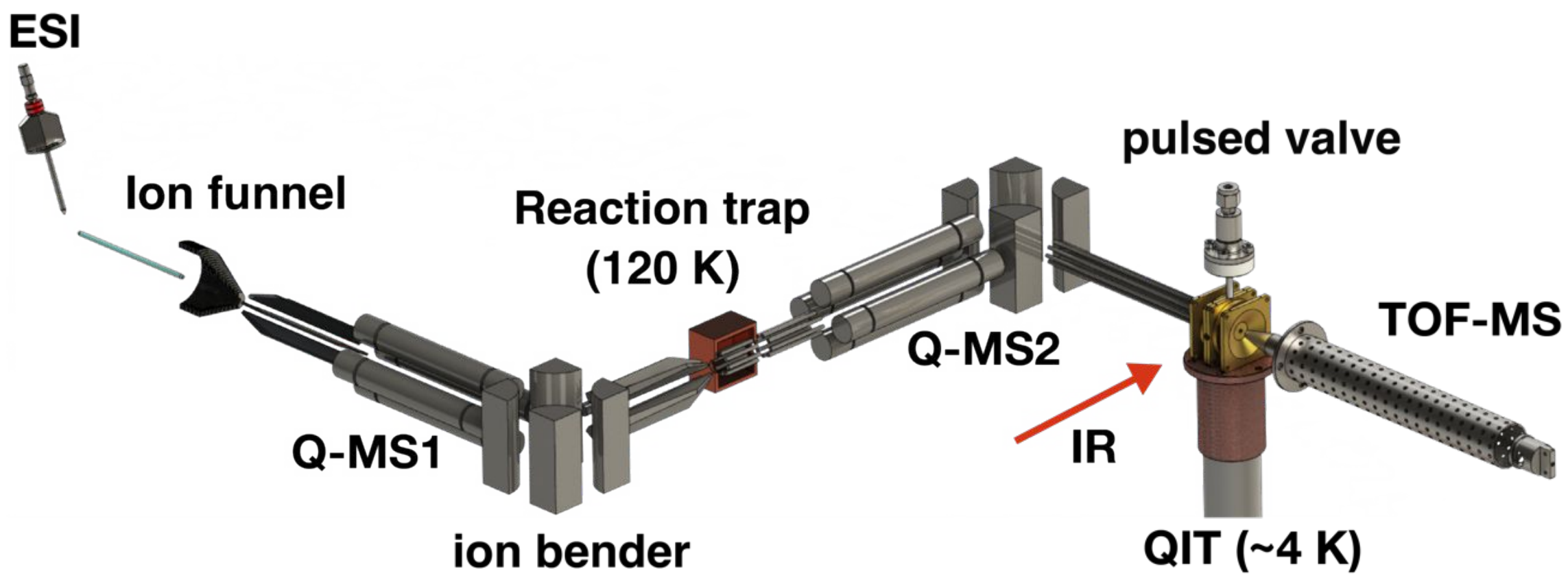

Figure S1. Experimental setup

Table S1. The binding energies ΔG_{298} (kJ mol^{-1}) of 12C4, 15C5, and 18C6 with alkali metal ions, defined as the Gibbs free energy difference between the complex and the separated ion and crown ether, calculated at the B3LYP/6-311++G(d,p) level of theory.

	12C4	15C5	18C6
Li	335	386	388
Na	233	293	310
K	159	195	251
Rb	117	146	190
Cs	94	117	148

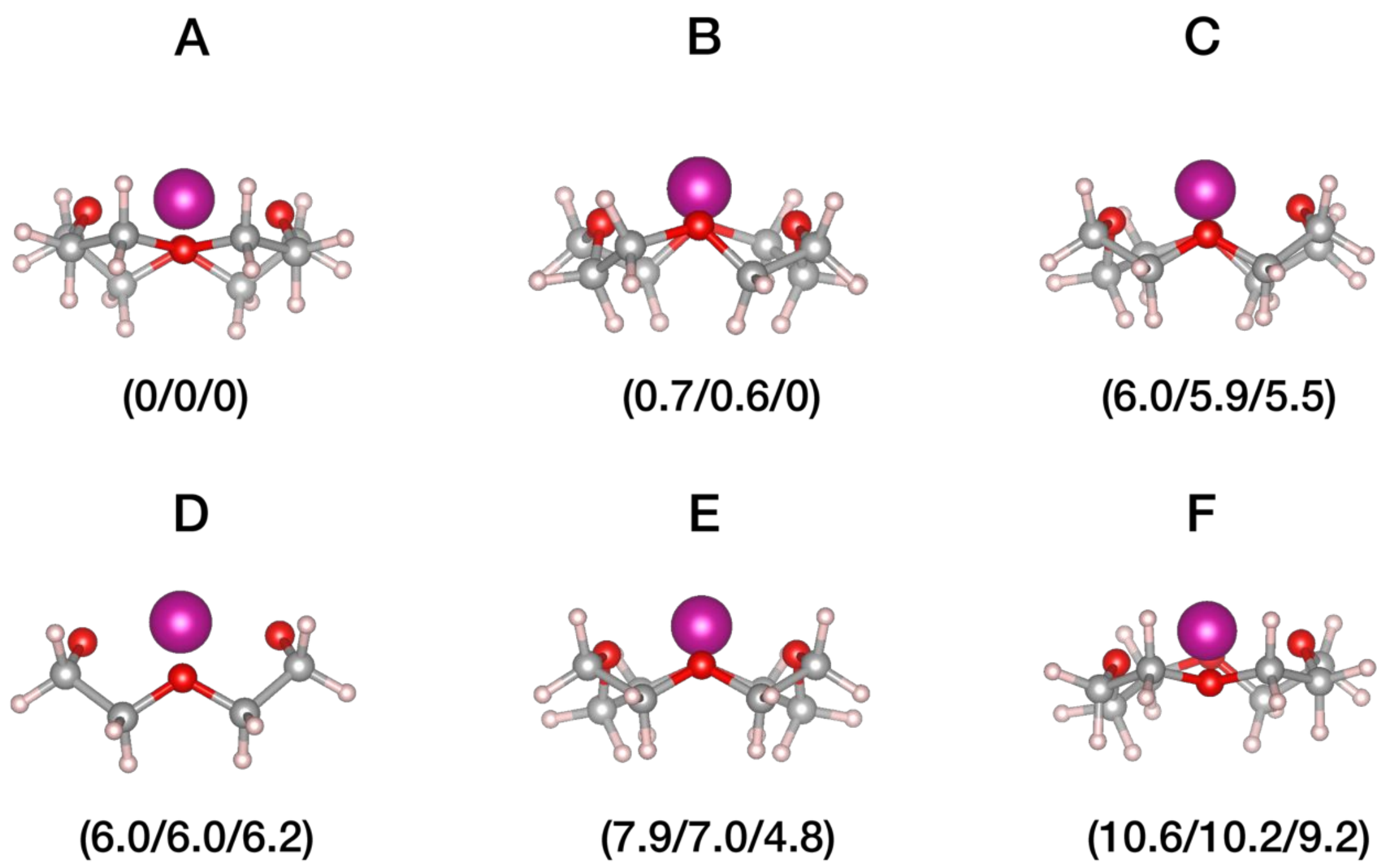

Figure S2. Low energy structures of the Li^+12C4 complex calculated at B3LYP/6-311++G(d,p) level. The relative zero-point corrected energies and Gibbs free energies (kJ mol^{-1}) at 120 K/298 K are given for each conformer.

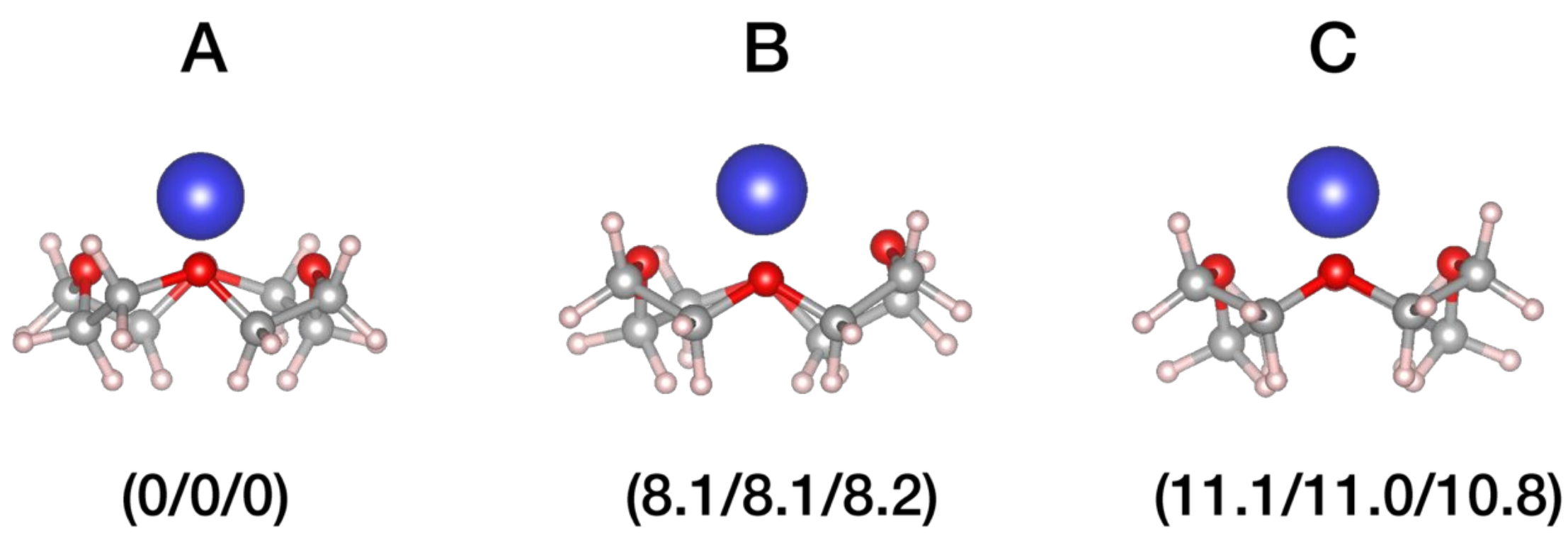

Figure S3. Low energy structures of the Na^+12C4 complex calculated at B3LYP/6-311++G(d,p) level. The relative zero-point corrected energies and Gibbs free energies (kJ mol^{-1}) at 120 K/298 K are given for each conformer.

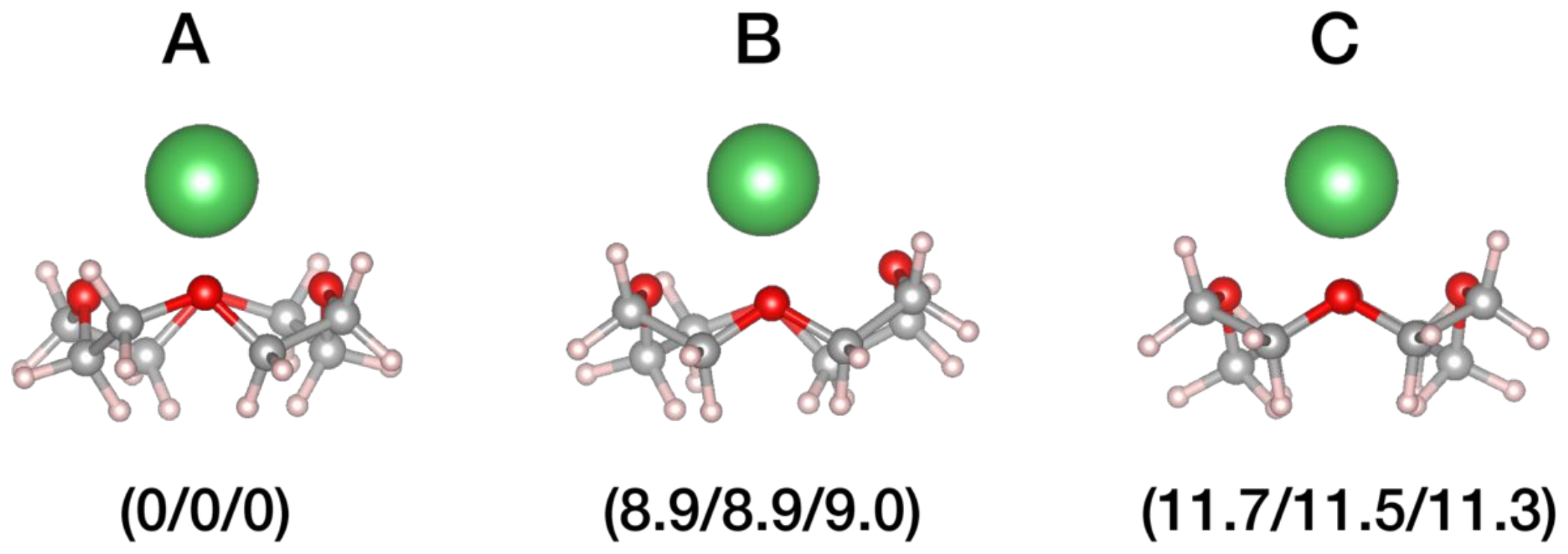

Figure S4. Low energy structures of the K^+12C4 complex calculated at B3LYP/6-311++G(d,p) level. The relative zero-point corrected energies and Gibbs free energies (kJ mol^{-1}) at 120 K/298 K are given for each conformer.

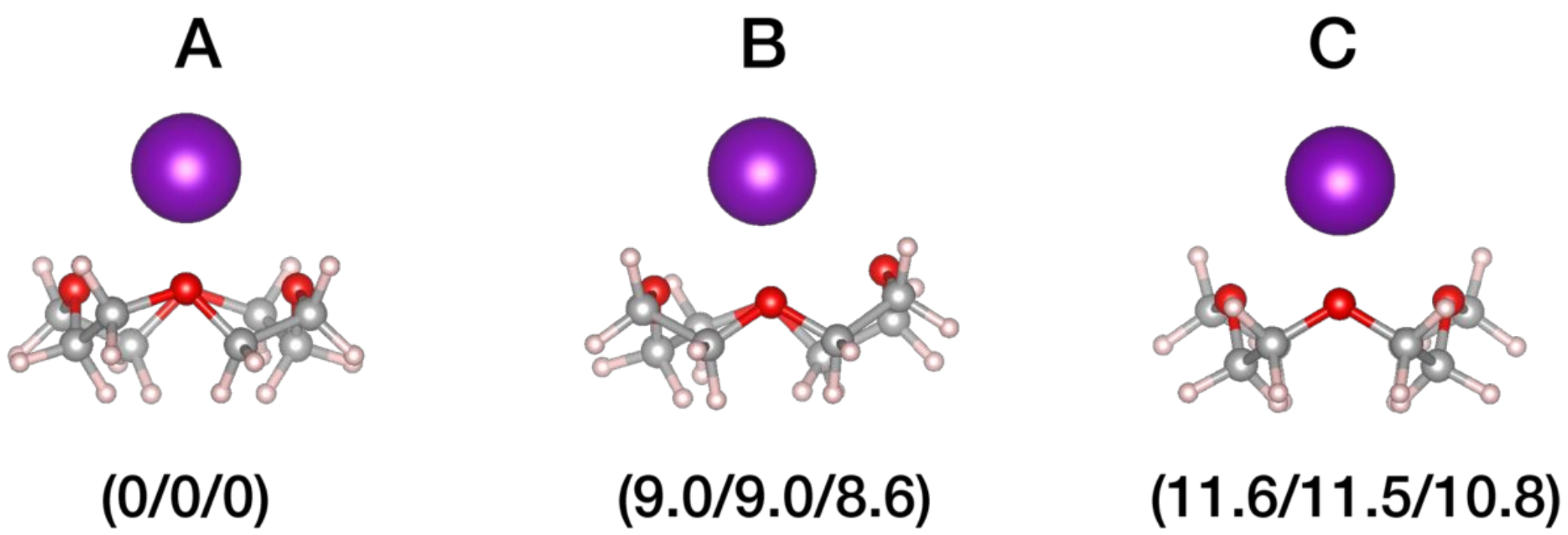

Figure S5. Low energy structures of the Rb^+12C4 complex calculated at B3LYP/6-311++G(d,p) level. The relative zero-point corrected energies and Gibbs free energies (kJ mol^{-1}) at 120 K/298 K are given for each conformer.

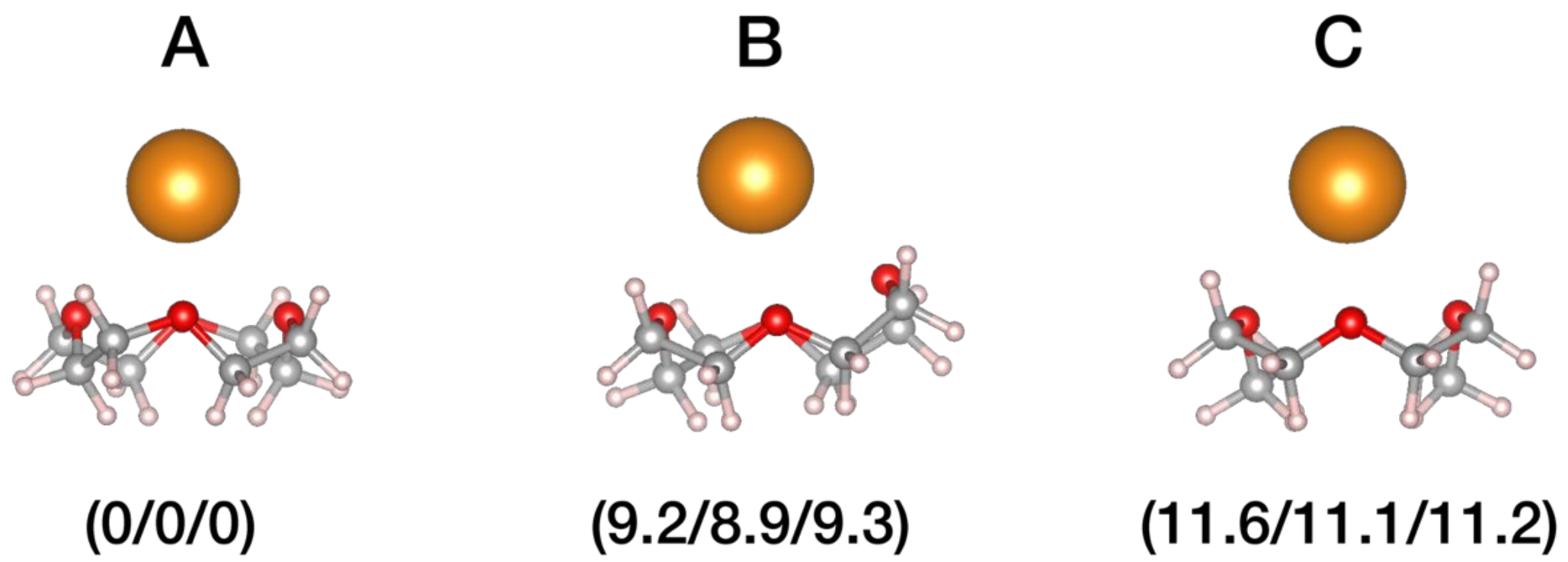

Figure S6. Low energy structures of the Cs^+12C4 complex calculated at B3LYP/6-311++G(d,p) level. The relative zero-point corrected energies and Gibbs free energies (kJ mol^{-1}) at 120 K/298 K are given for each conformer.

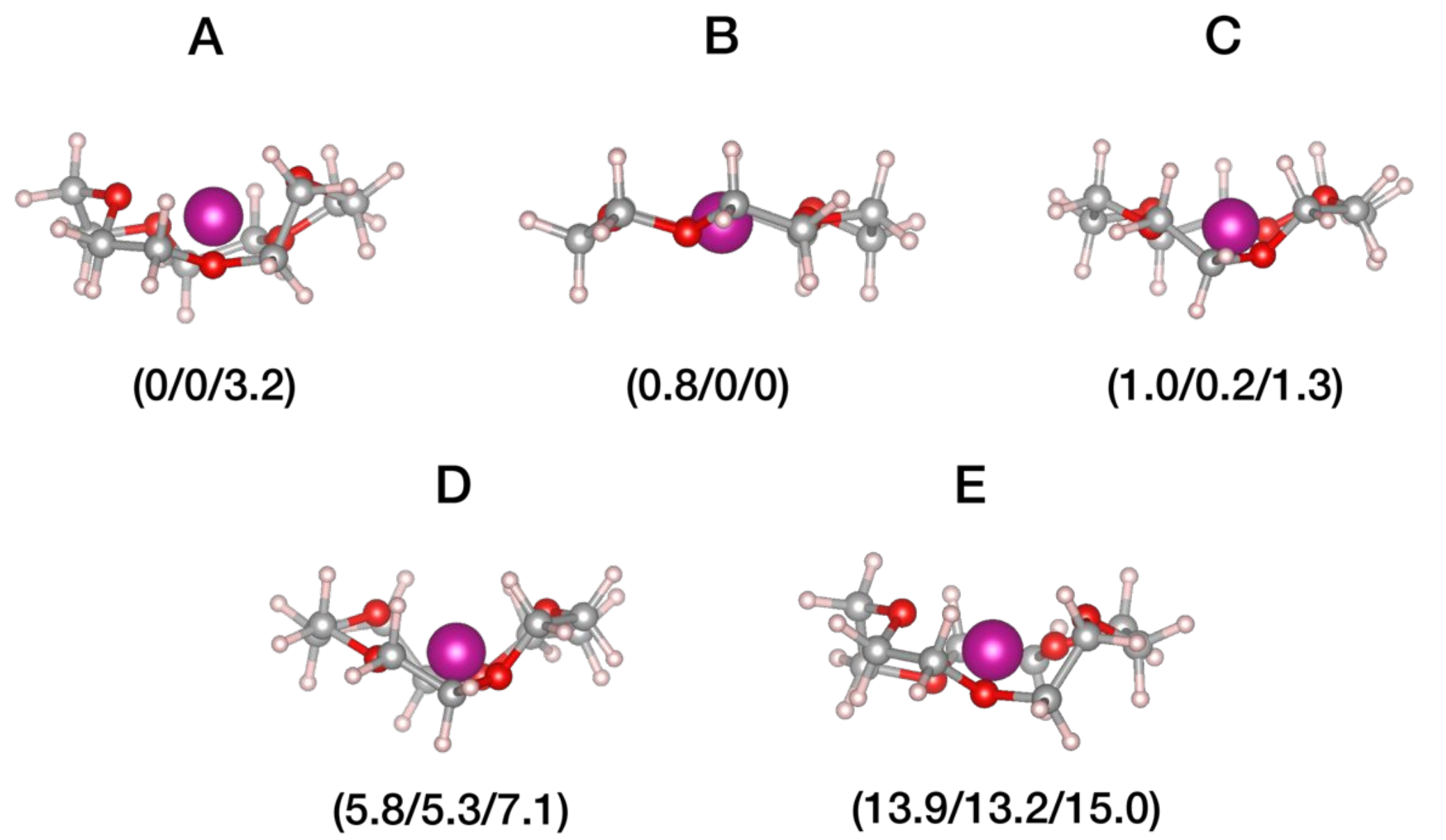

Figure S7. Low energy structures of the Li^+15C5 complex calculated at B3LYP/6-311++G(d,p) level. The relative zero-point corrected energies and Gibbs free energies (kJ mol^{-1}) at 120 K/298 K are given for each conformer.

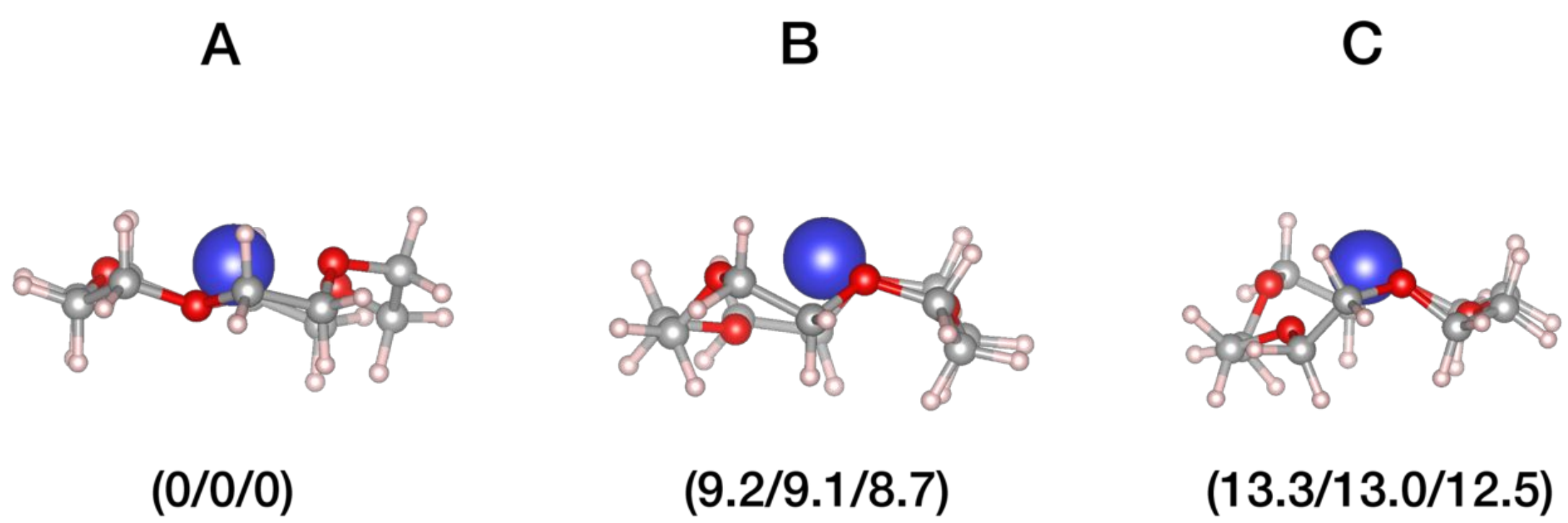

Figure S8. Low energy structures of the Na^+15C5 complex calculated at B3LYP/6-311++G(d,p) level. The relative zero-point corrected energies and Gibbs free energies (kJ mol^{-1}) at 120 K/298 K are given for each conformer.

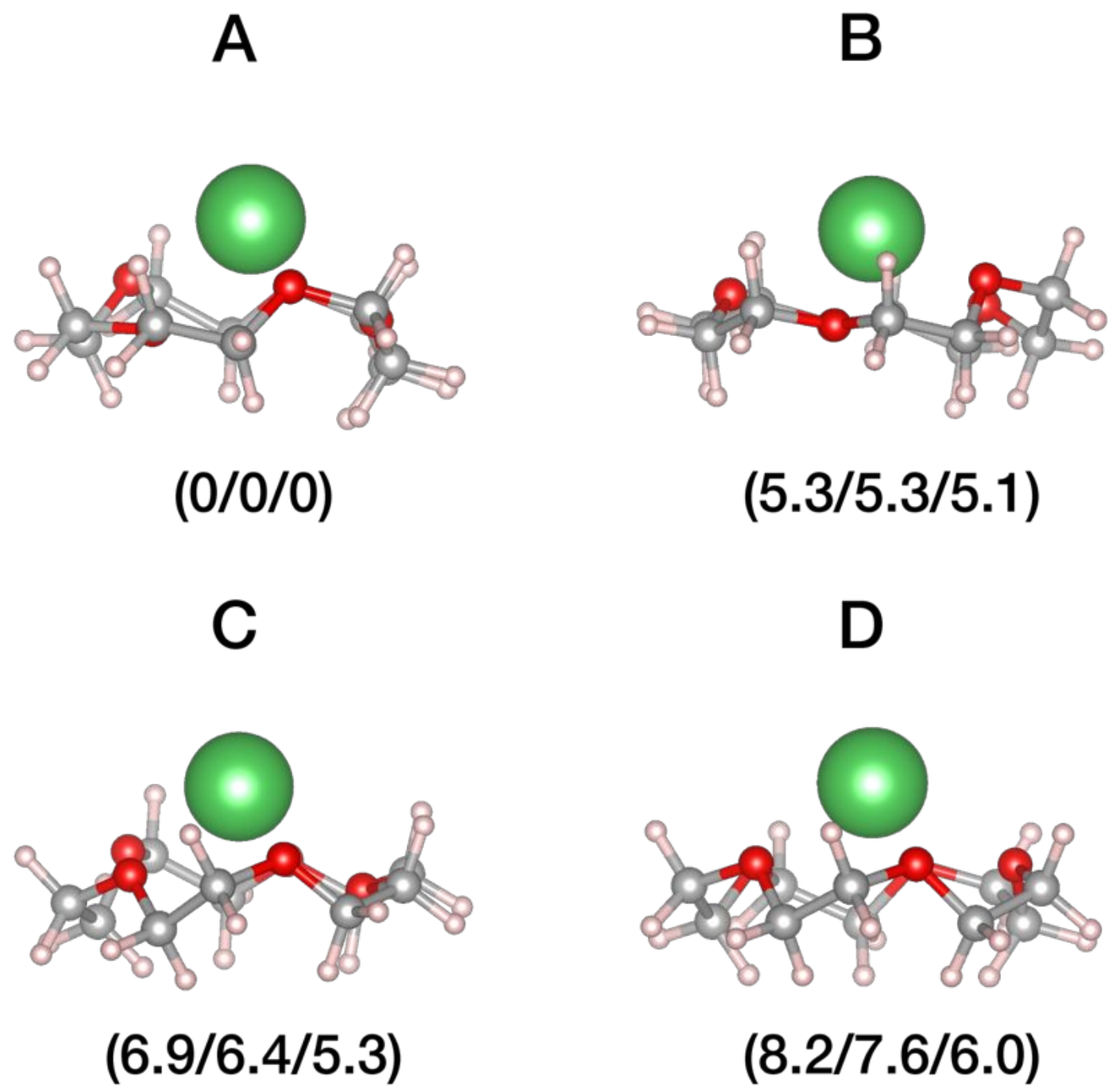

Figure S9. Low energy structures of the K^+15C5 complex calculated at B3LYP/6-311++G(d,p) level. The relative zero-point corrected energies and Gibbs free energies (kJ mol^{-1}) at 120 K/298 K are given for each conformer.

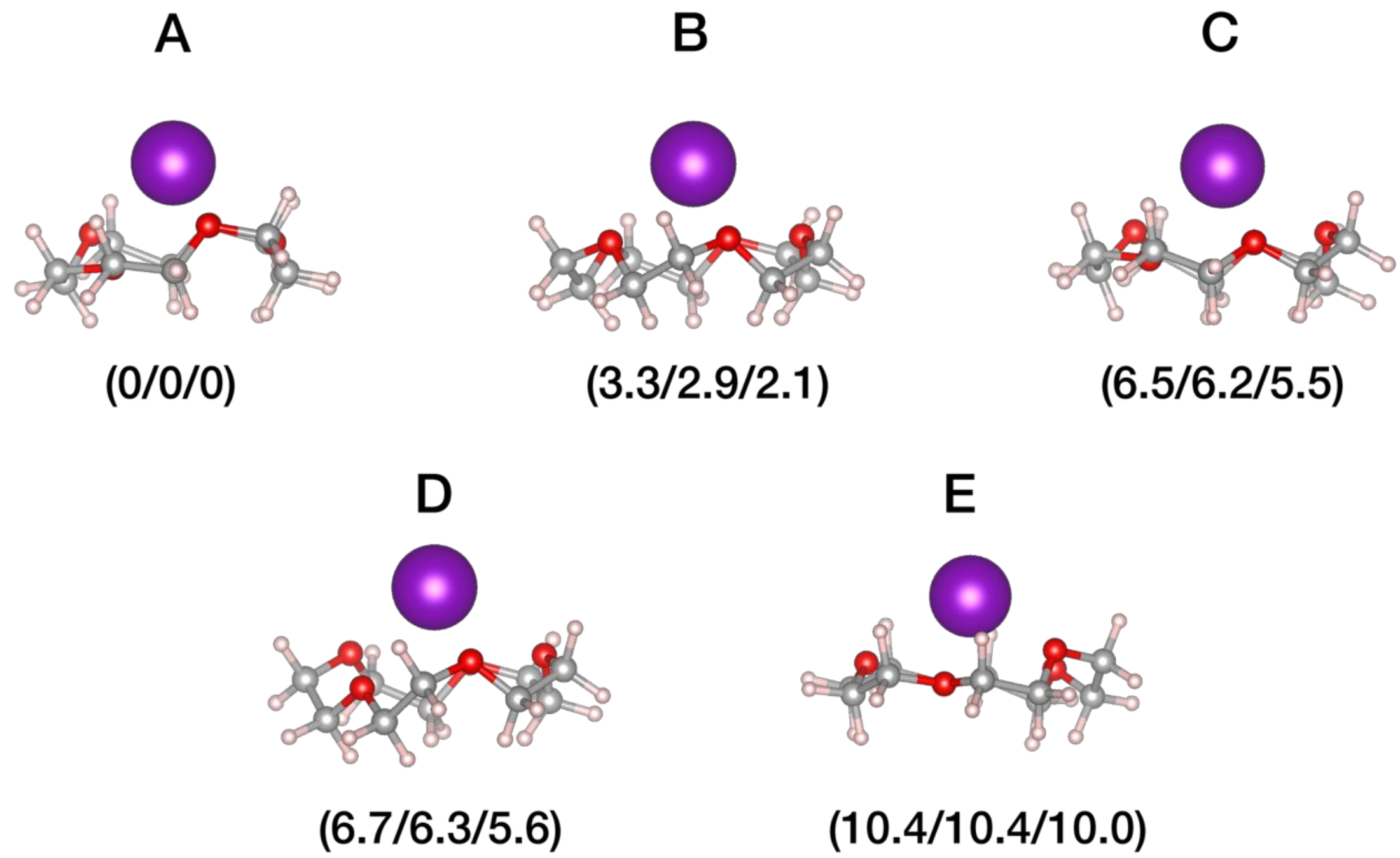

Figure S10. Low energy structures of the Rb^+15C5 complex calculated at B3LYP/6-311++G(d,p) level. The relative zero-point corrected energies and Gibbs free energies (kJ mol^{-1}) at 120 K/298 K are given for each conformer.

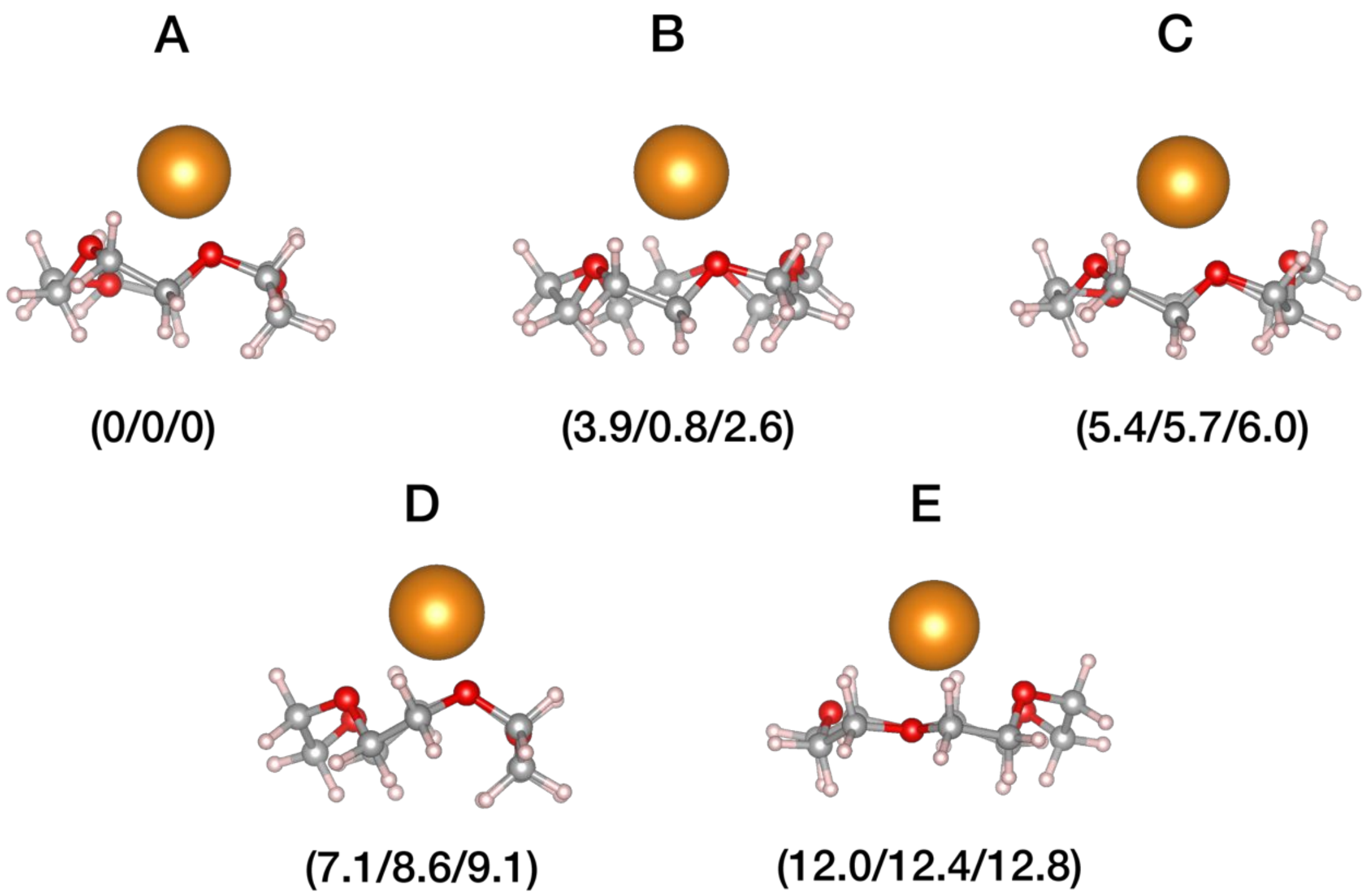

Figure S11. Low energy structures of the Cs^+15C5 complex calculated at B3LYP/6-311++G(d,p) level. The relative zero-point corrected energies and Gibbs free energies (kJ mol^{-1}) at 120 K/298 K are given for each conformer.

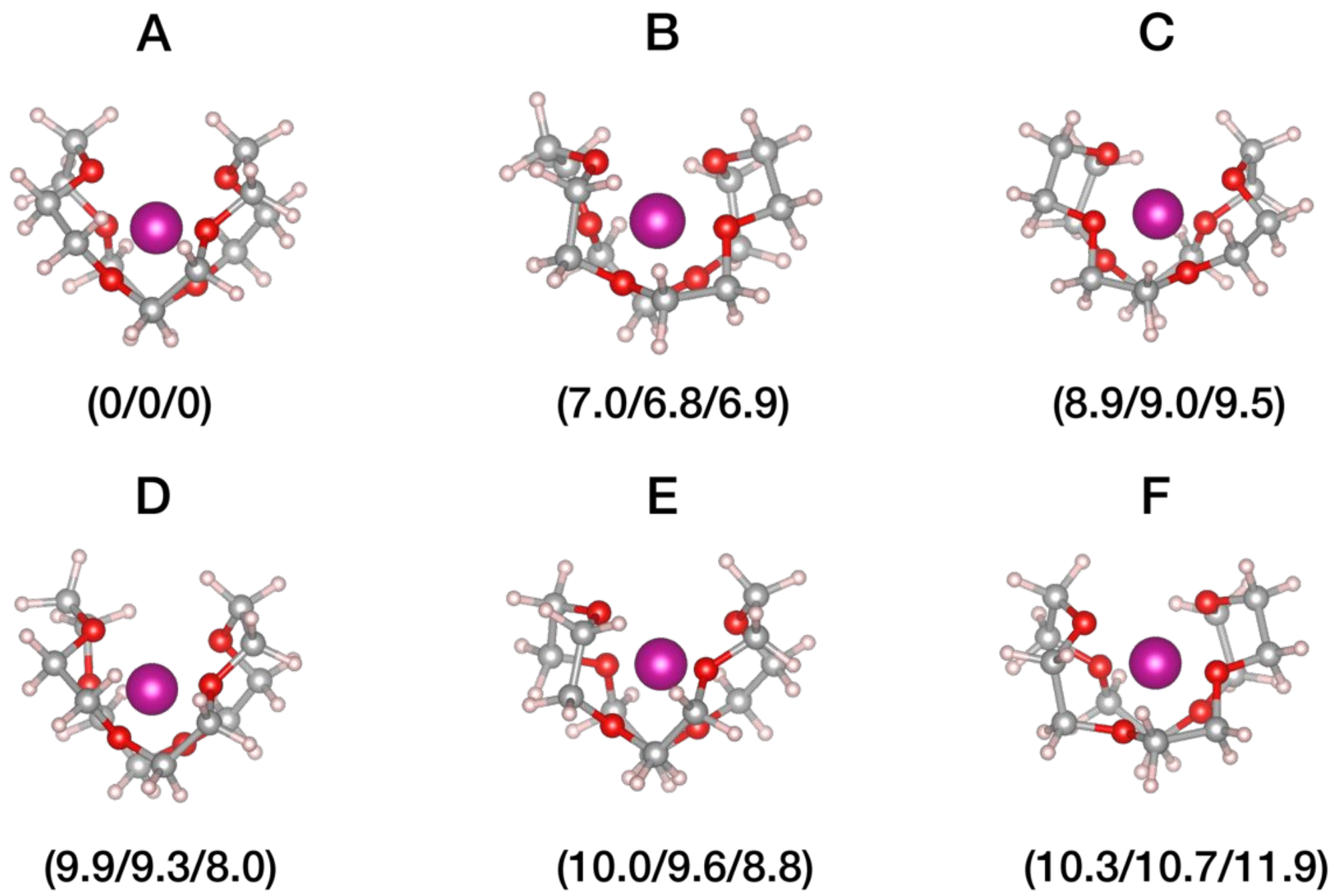

Figure S12. Low energy structures of the Li^+18C6 complex calculated at B3LYP/6-311++G(d,p) level. The relative zero-point corrected energies and Gibbs free energies (kJ mol^{-1}) at 120 K/298 K are given for each conformer.

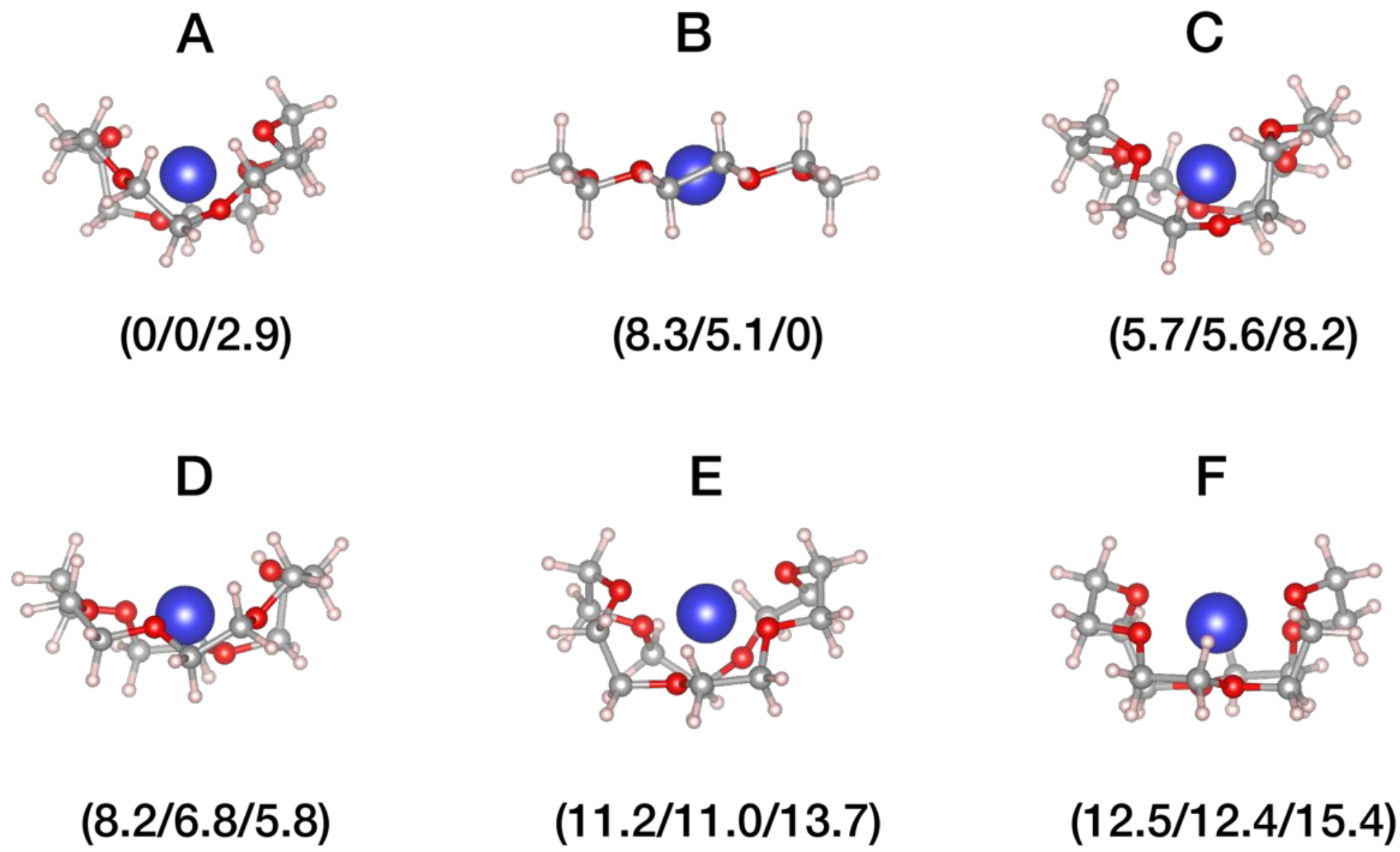

Figure S13. Low energy structures of the Na^+18C6 complex calculated at B3LYP/6-311++G(d,p) level. The relative zero-point corrected energies and Gibbs free energies (kJ mol^{-1}) at 120 K/298 K are given for each conformer.

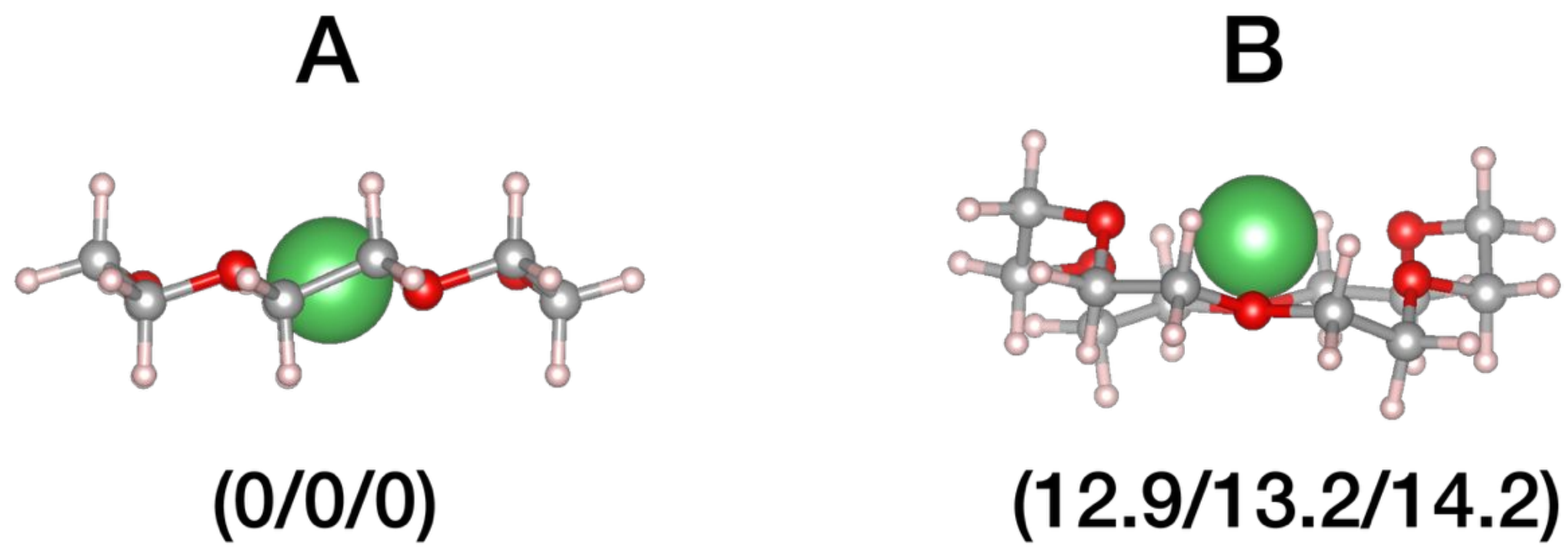

Figure S14. Low energy structures of the K^{+}18C6 complex calculated at B3LYP/6-311++G(d,p) level. The relative zero-point corrected energies and Gibbs free energies (kJ mol^{-1}) at 120 K/298 K are given for each conformer.

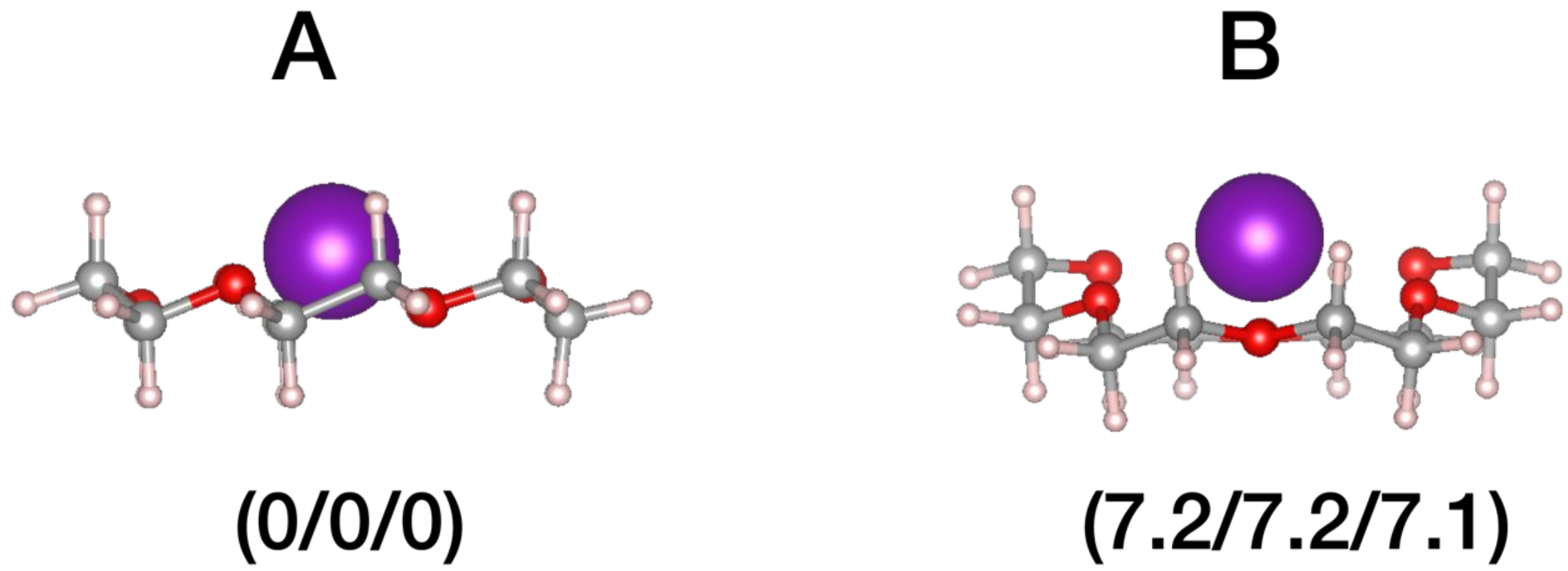

Figure S15. Low energy structures of the Rb^{+}18C6 complex calculated at B3LYP/6-311++G(d,p) level. The relative zero-point corrected energies and Gibbs free energies (kJ mol^{-1}) at 120 K/298 K are given for each conformer.

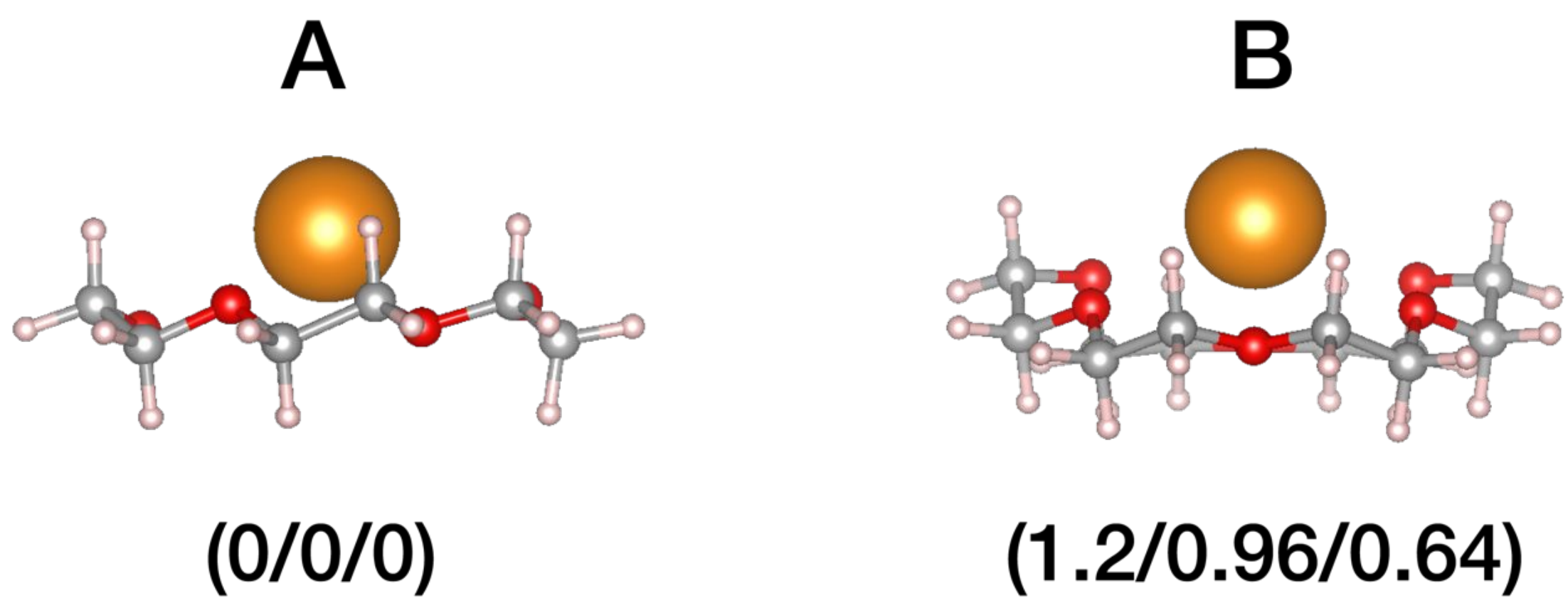

Figure S16. Low energy structures of the Cs^{+}18C6 complex calculated at B3LYP/6-311++G(d,p) level. The relative zero-point corrected energies and Gibbs free energies (kJ mol^{-1}) at 120 K/298 K are given for each conformer.

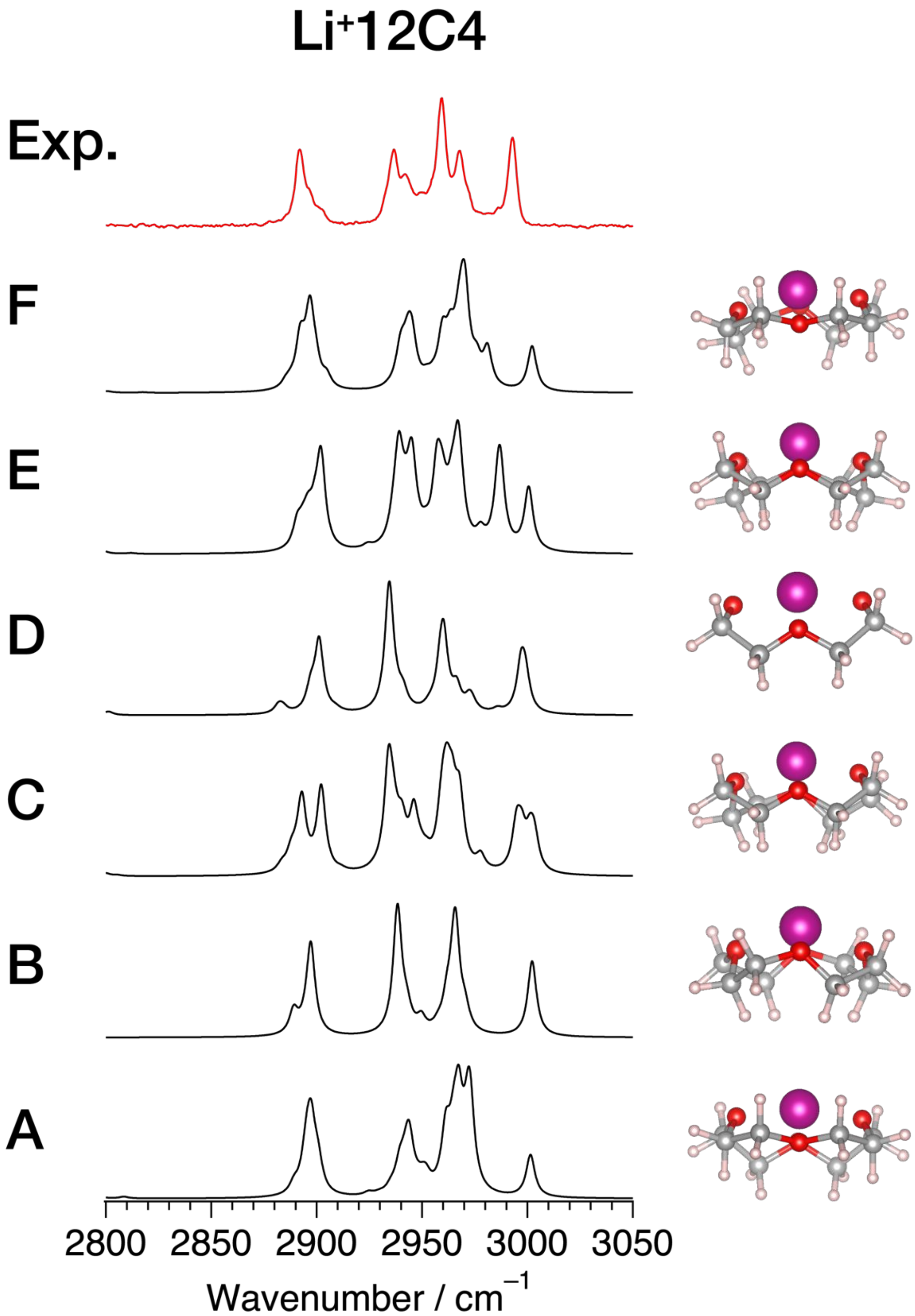

Figure S17. Comparison of experimental and calculated anharmonic spectra of Li^+12C4. The structures are the same as those shown in Figure S2. The relative Gibbs free energies (kJ mol^{-1}) at 298 K are given for each conformer.

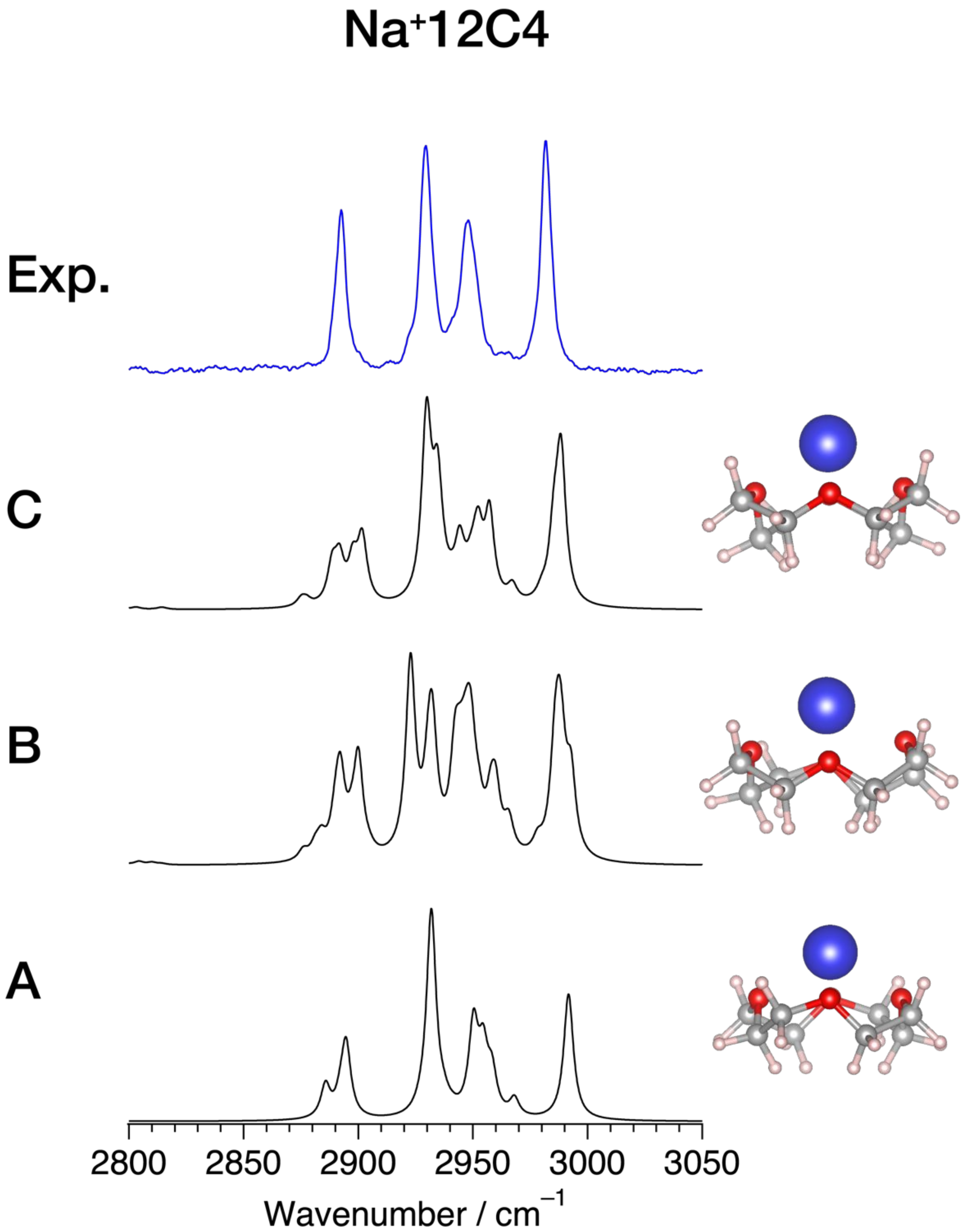

Figure S18. Comparison of experimental and calculated anharmonic spectra of Na^+12C4. The structures are the same as those shown in Figure S3. The relative zero-point corrected energies and Gibbs free energies (kJ mol^{-1}) at 120 K/298 K are given for each conformer.

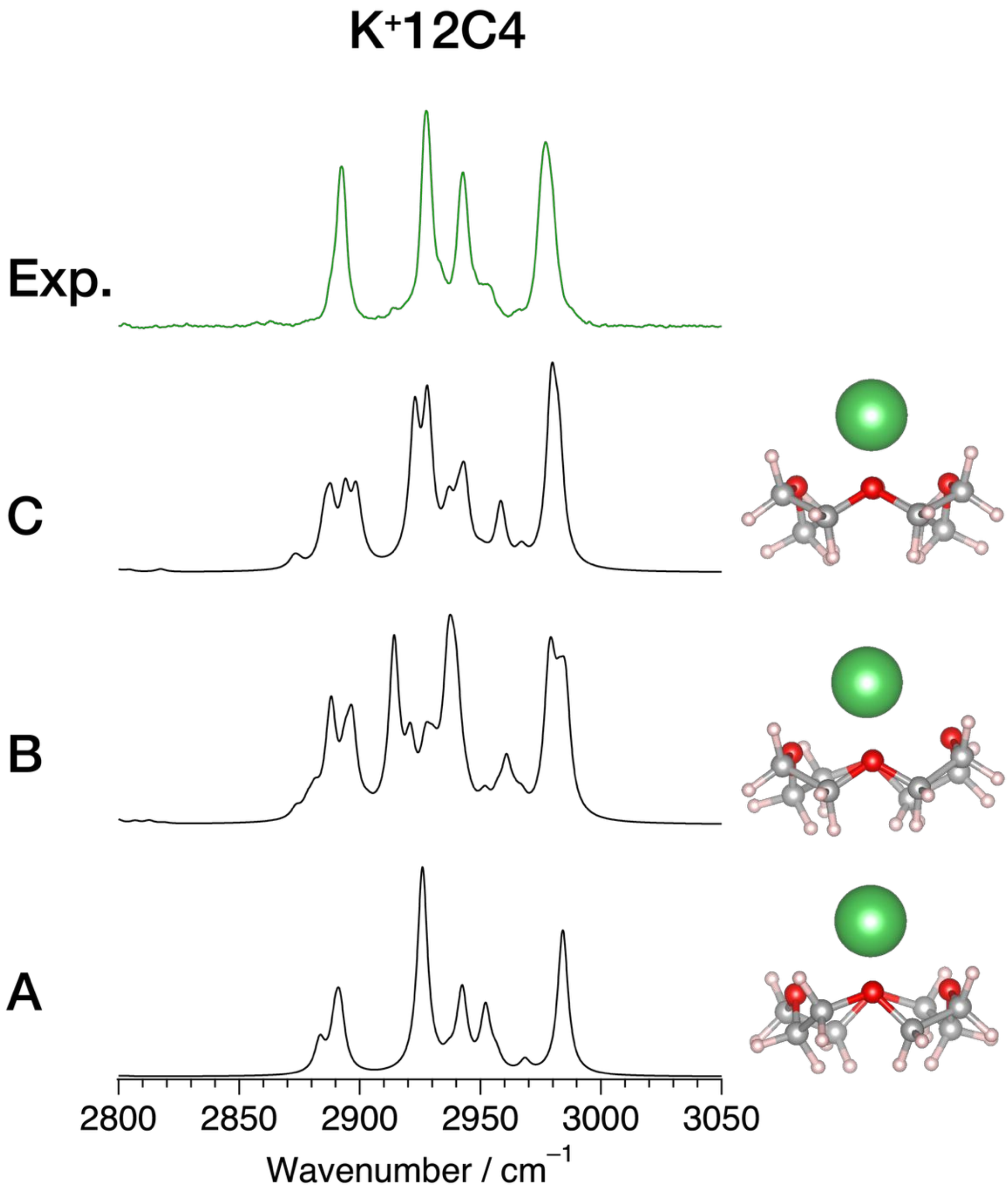

Figure S19. Comparison of experimental and calculated anharmonic spectra of K^+12C4. The structures are the same as those shown in Figure S4. The relative zero-point corrected energies and Gibbs free energies (kJ mol^{-1}) at 120 K/298 K are given for each conformer.

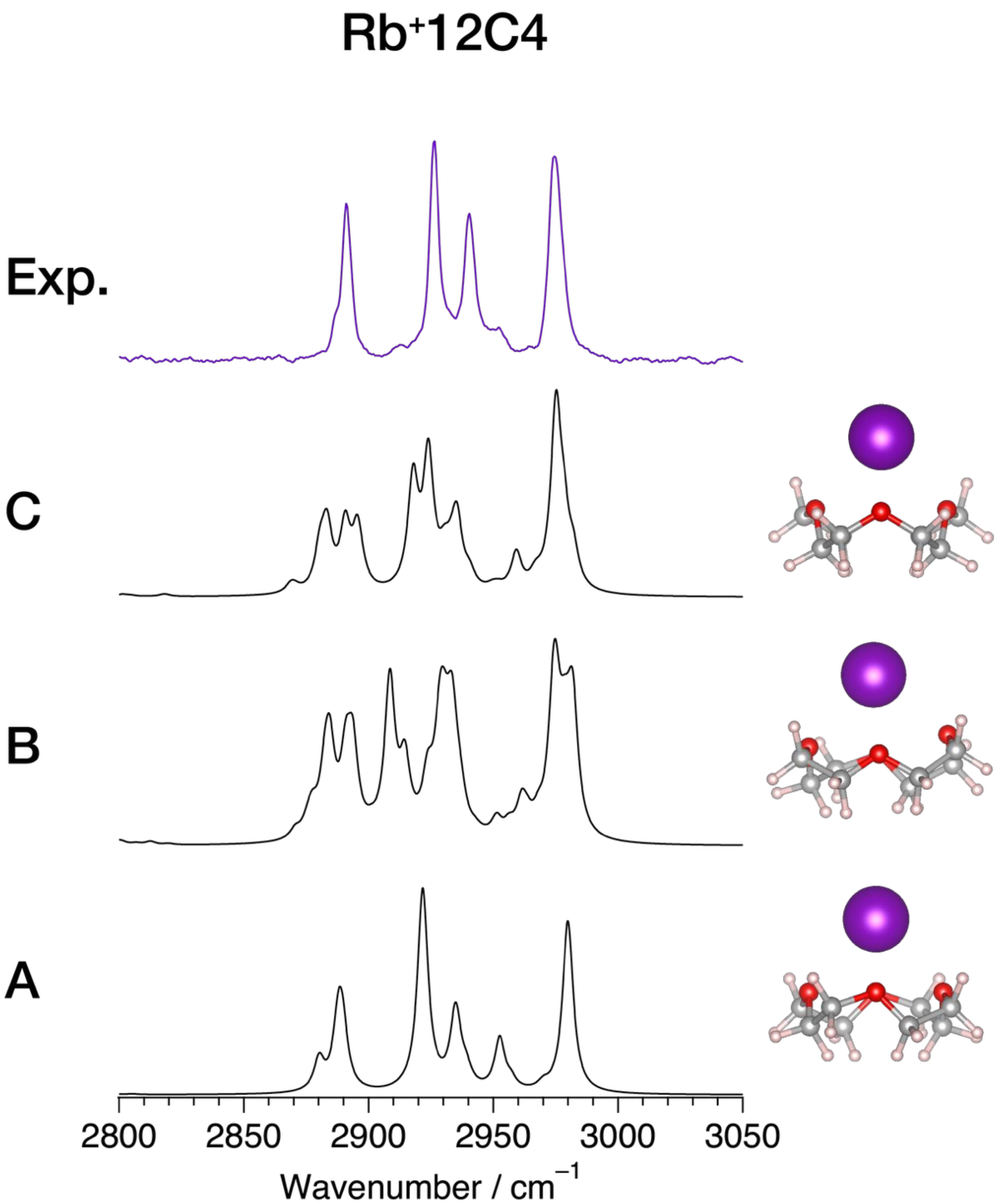

Figure S20. Comparison of experimental and calculated anharmonic spectra of Rb^{+}12C4. The structures are the same as those shown in Figure S5. The relative zero-point corrected energies and Gibbs free energies (kJ mol^{-1}) at 120 K/298 K are given for each conformer.

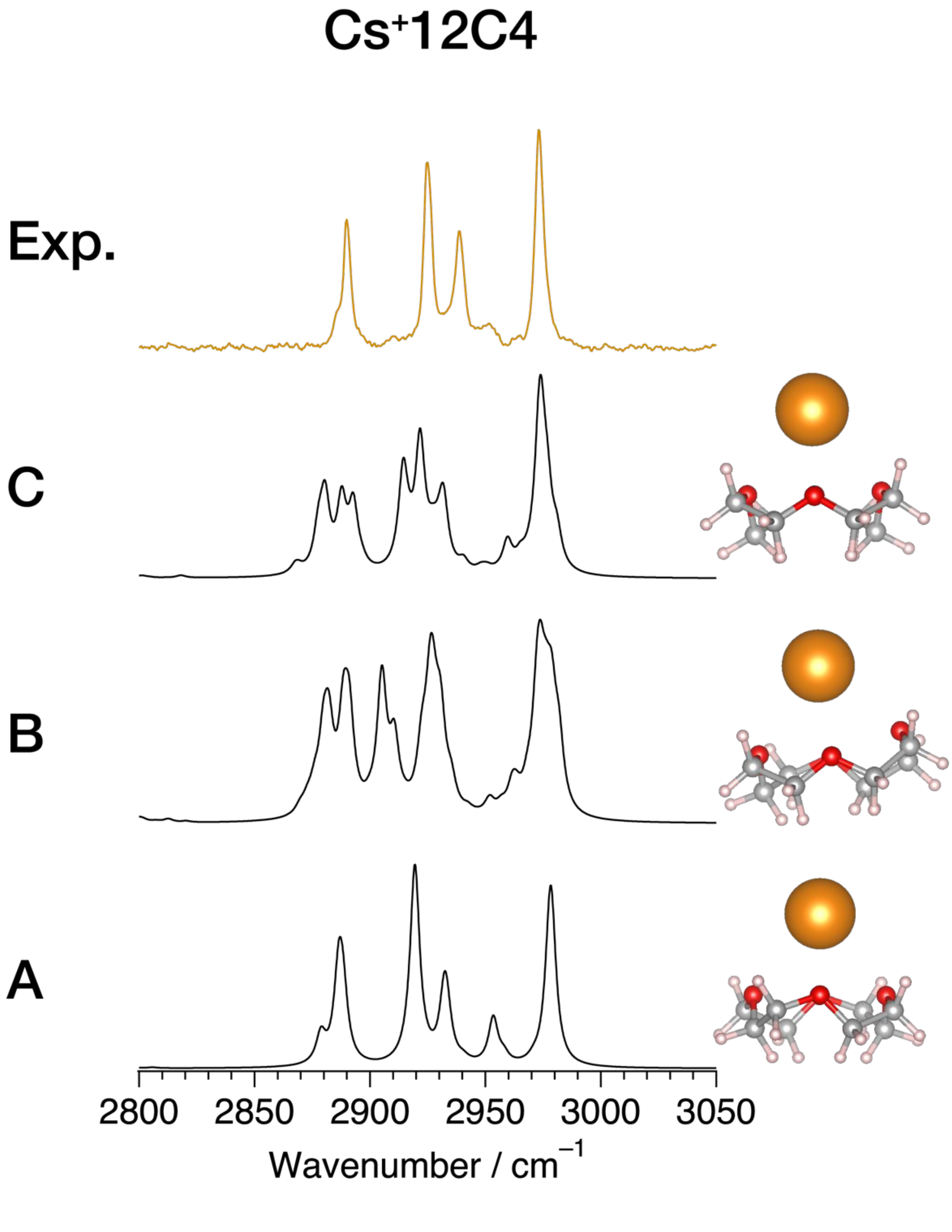

Figure S21. Comparison of experimental and calculated anharmonic spectra of Cs^+12C4. The structures are the same as those shown in Figure S6. The relative zero-point corrected energies and Gibbs free energies (kJ mol^{-1}) at 120 K/298 K are given for each conformer.

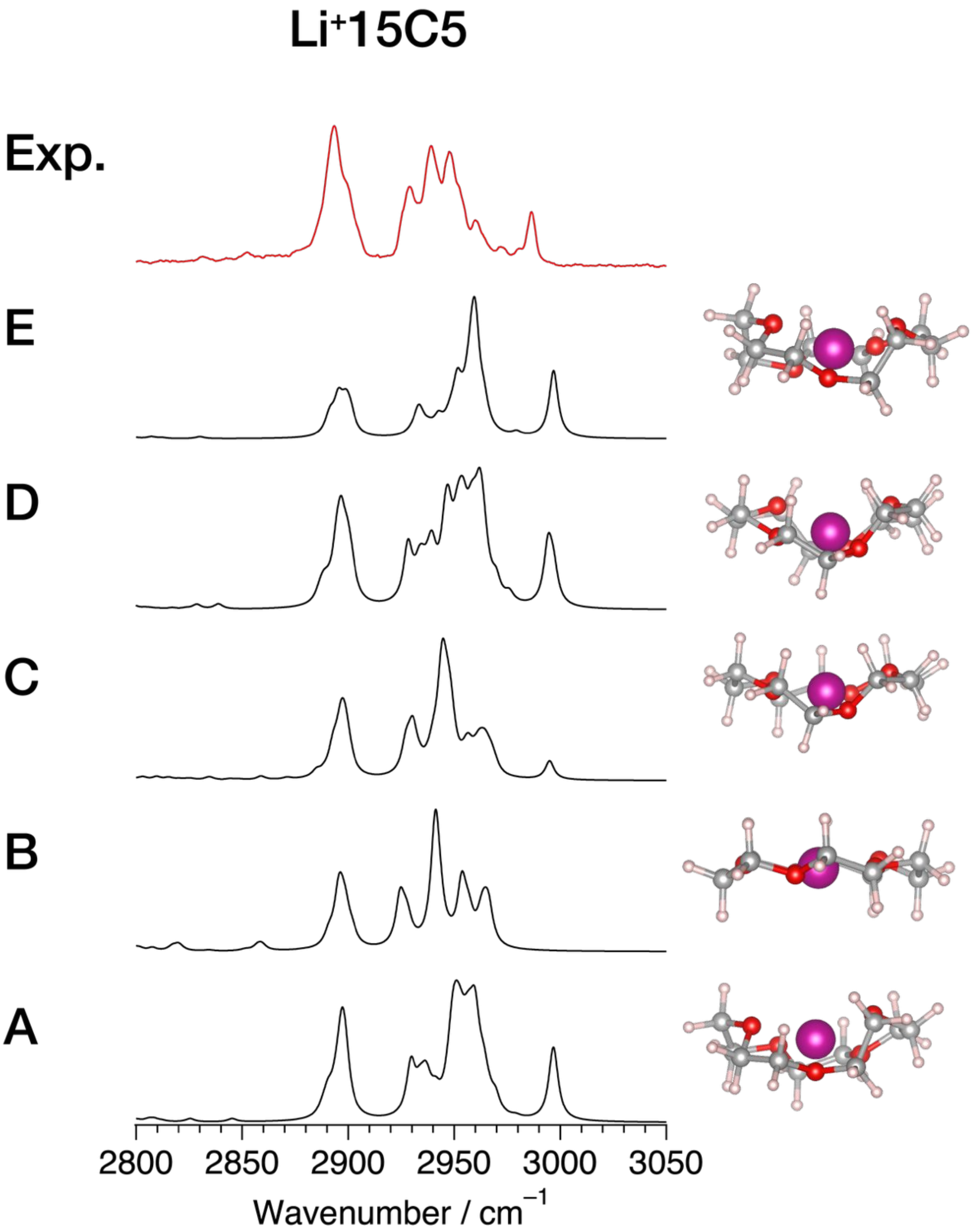

Figure S22. Comparison of experimental and calculated anharmonic spectra of Li^+15C5. The structures are the same as those shown in Figure S7. The relative zero-point corrected energies and Gibbs free energies (kJ mol^{-1}) at 120 K/298 K are given for each conformer.

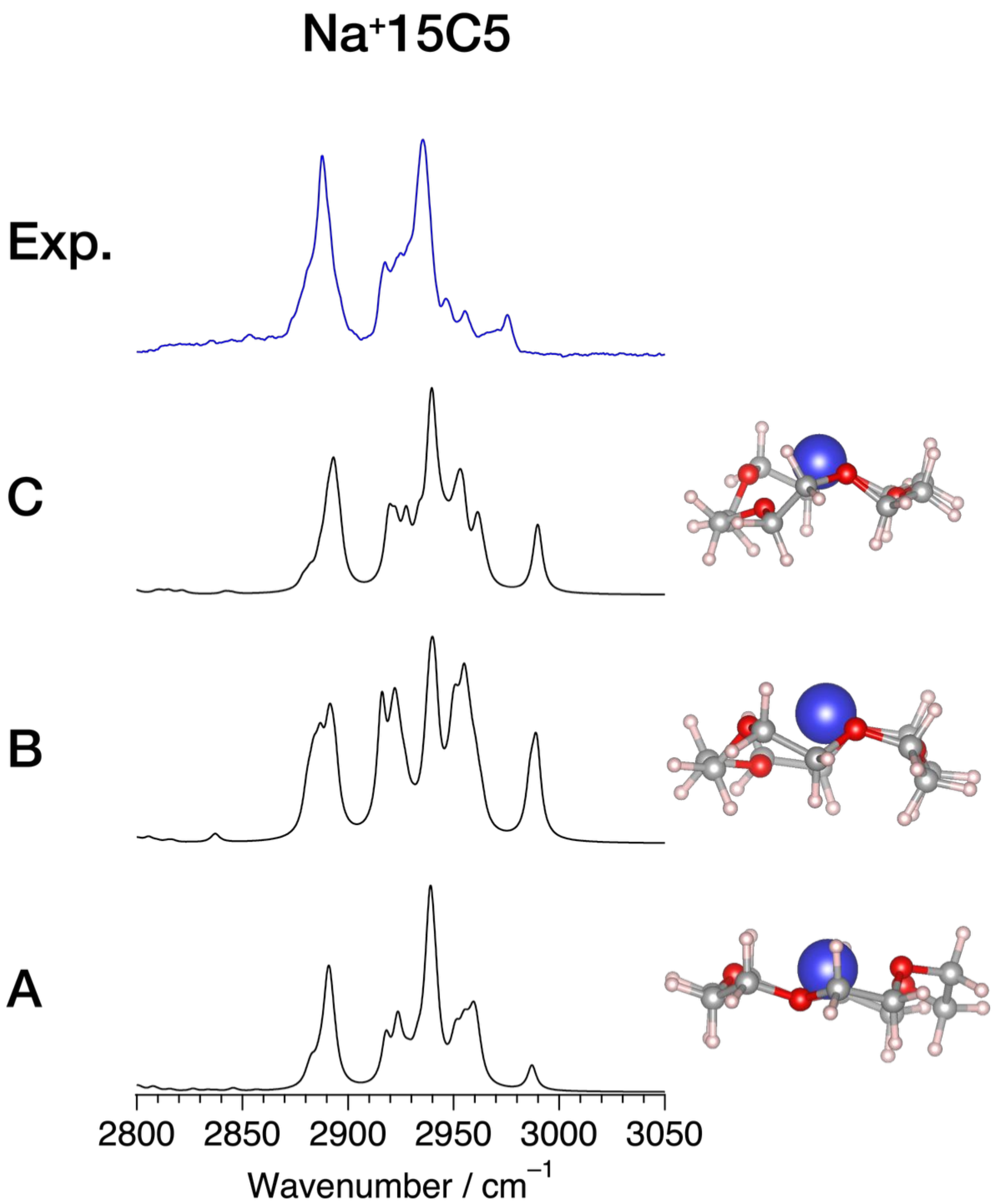

Figure S23. Comparison of experimental and calculated anharmonic spectra of Na^+15C5. The structures are the same as those shown in Figure S8. The relative zero-point corrected energies and Gibbs free energies (kJ mol^{-1}) at 120 K/298 K are given for each conformer.

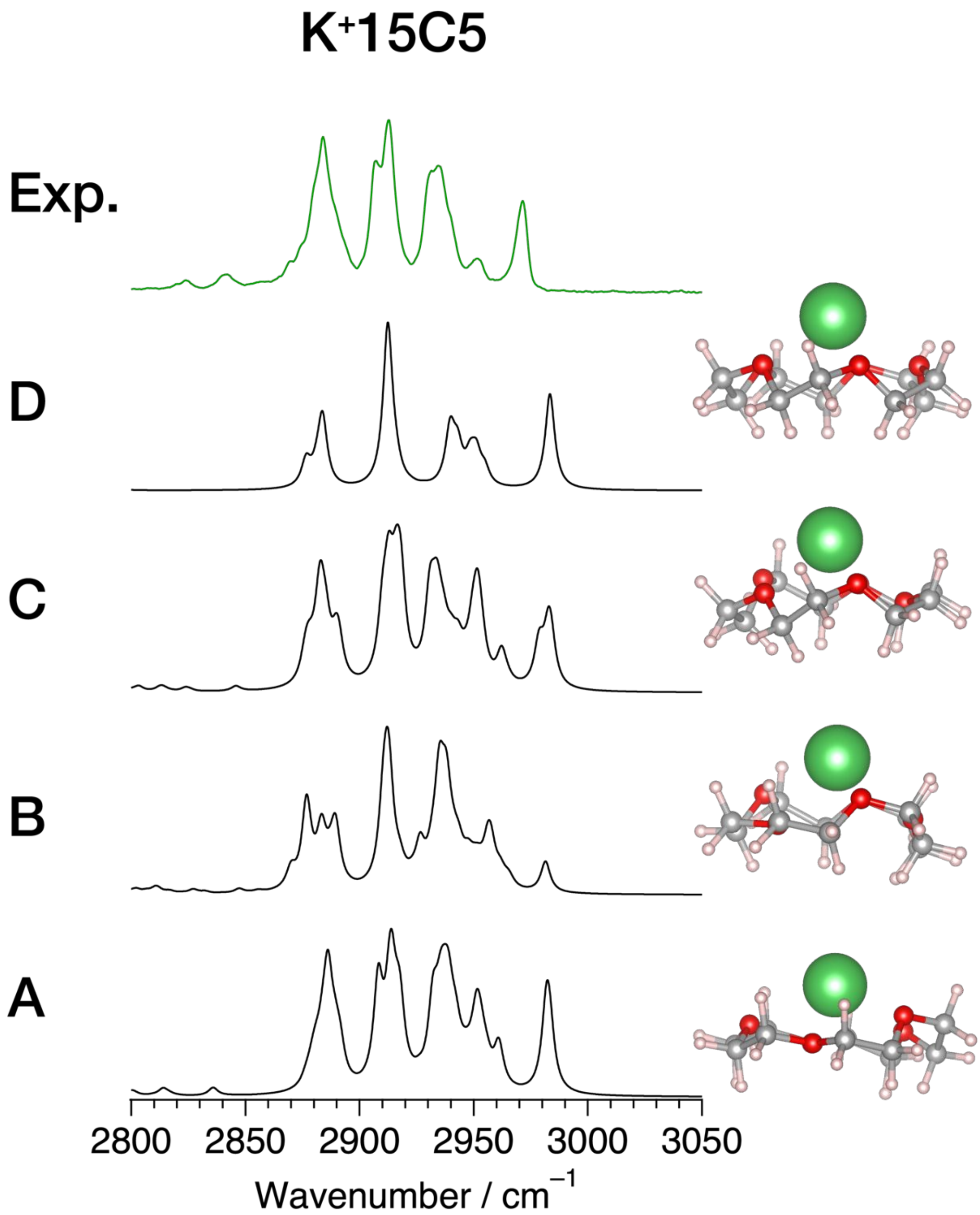

Figure S24. Comparison of experimental and calculated anharmonic spectra of K^+15C5. The structures are the same as those shown in Figure S9. The relative zero-point corrected energies and Gibbs free energies (kJ mol^{-1}) at 120 K/298 K are given for each conformer.

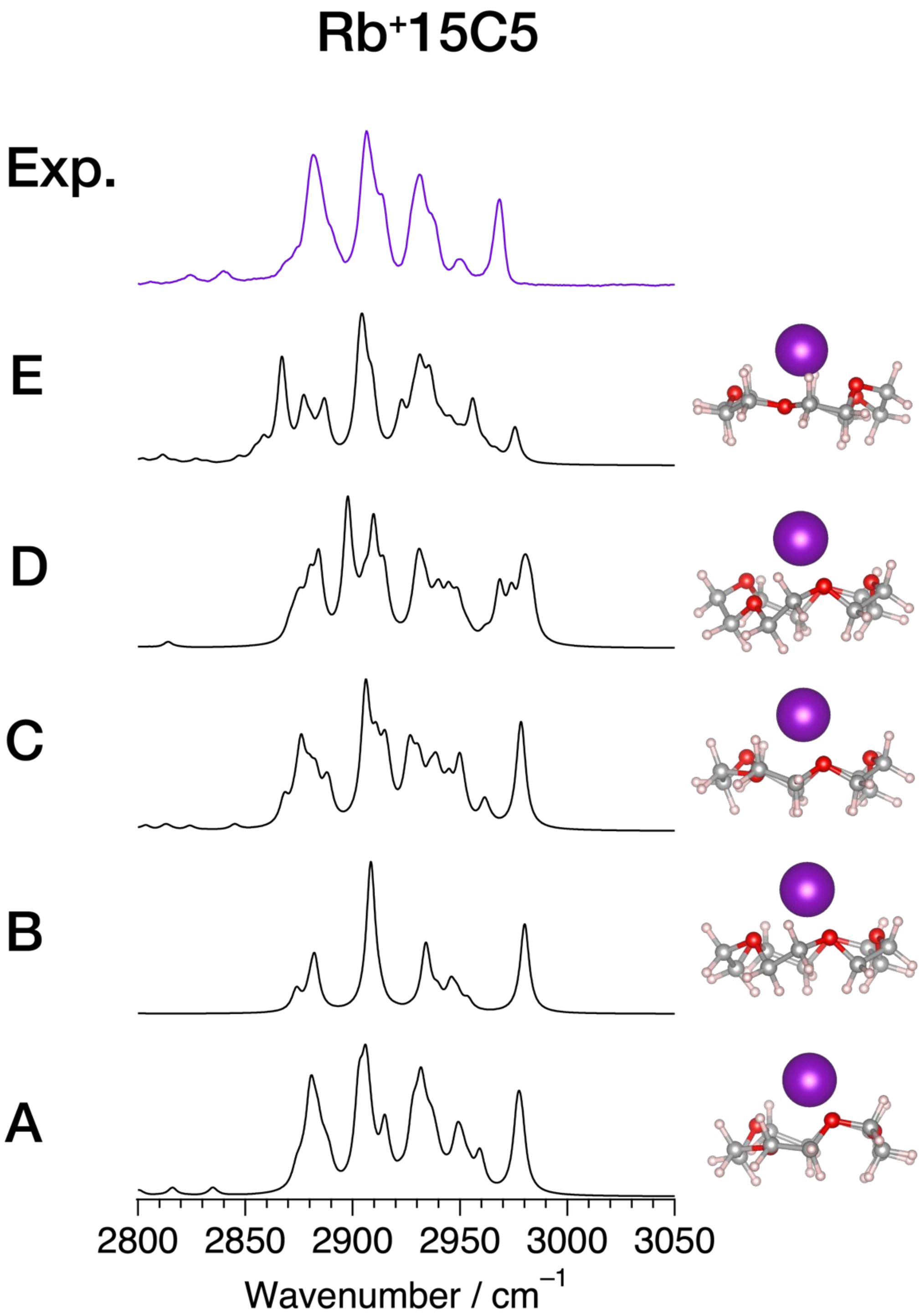

Figure S25. Comparison of experimental and calculated anharmonic spectra of Rb^+15C5. The structures are the same as those shown in Figure S10. The relative zero-point corrected energies and Gibbs free energies (kJ mol^{-1}) at 120 K/298 K are given for each conformer.

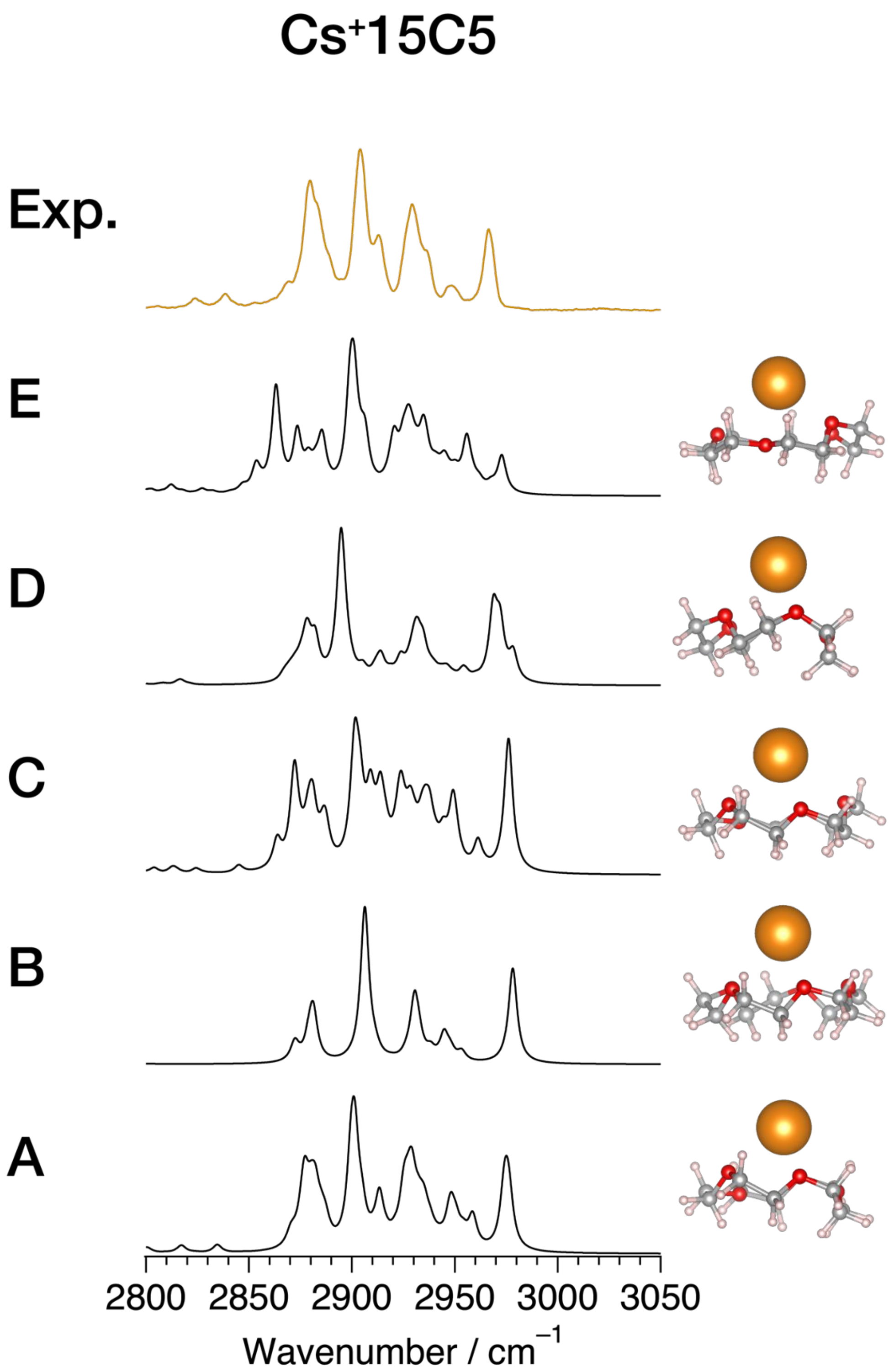

Figure S26. Comparison of experimental and calculated anharmonic spectra of Cs+15C5. The structures are the same as those shown in Figure S11. The relative zero-point corrected energies and Gibbs free energies (kJ mol^{-1}) at 120 K/298 K are given for each conformer.

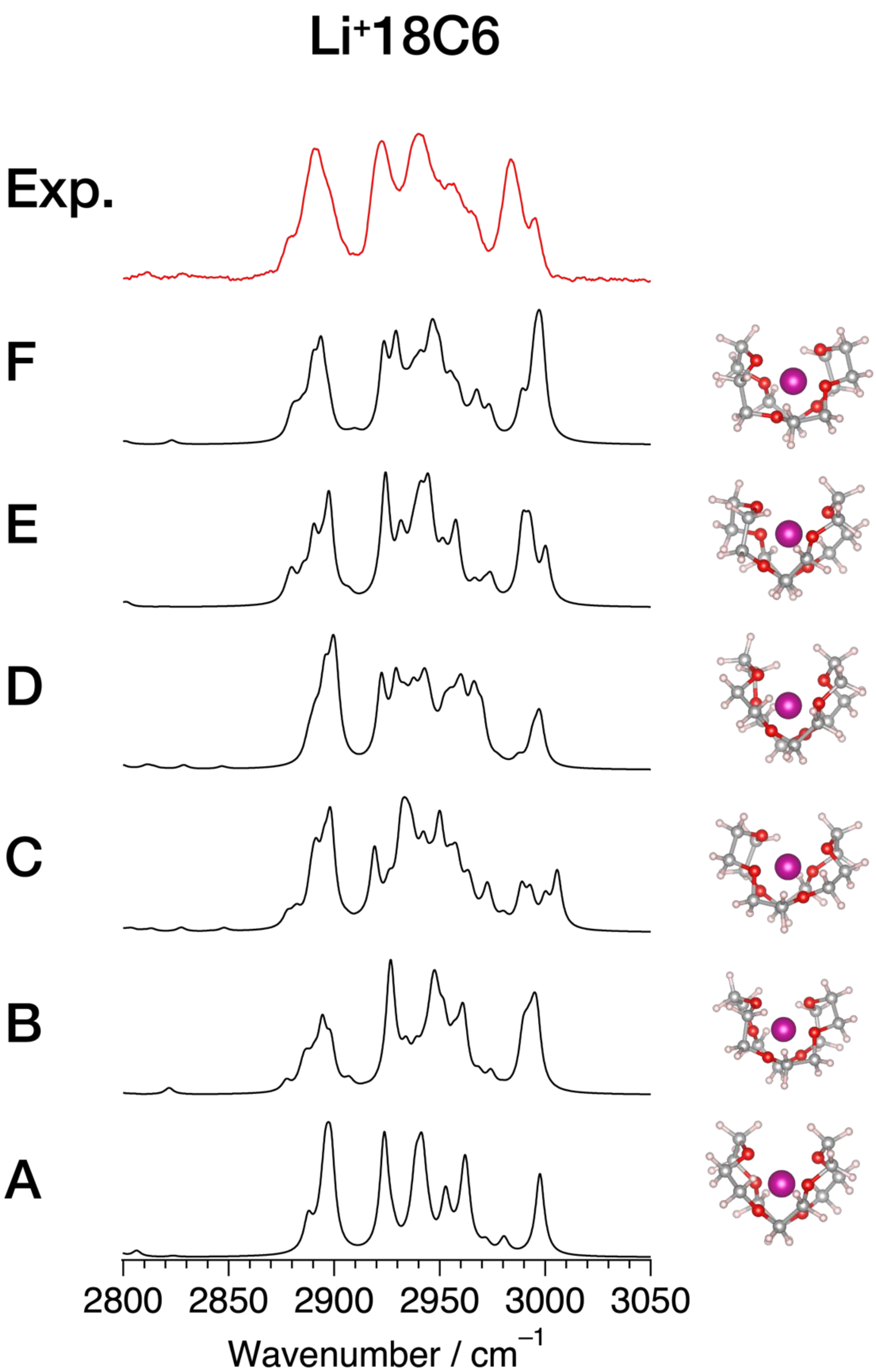

Figure S27. Comparison of experimental and calculated anharmonic spectra of Li^+18C6. The structures are the same as those shown in Figure S12. The relative zero-point corrected energies and Gibbs free energies (kJ mol^{-1}) at 120 K/298 K are given for each conformer.

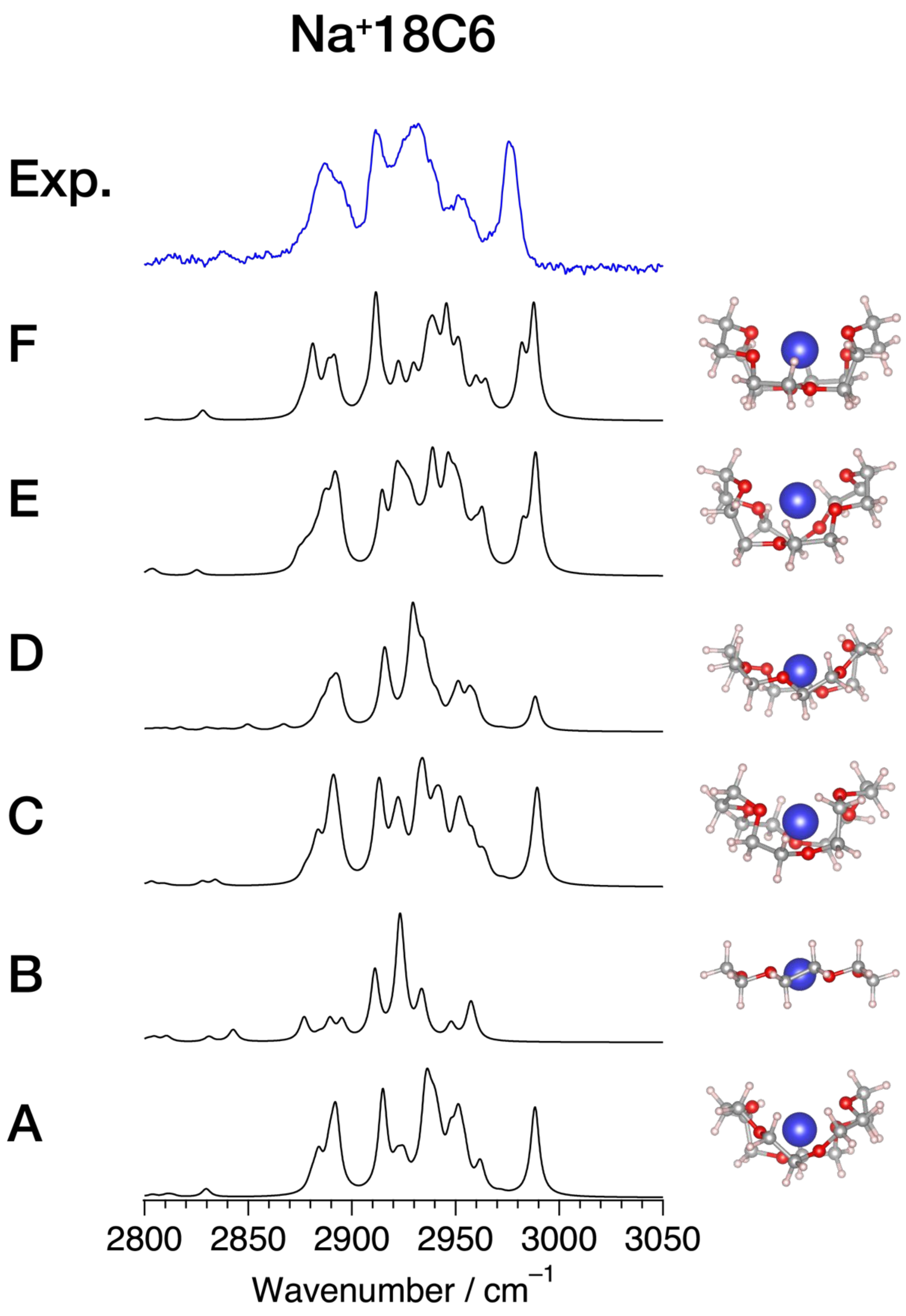

Figure S28. Comparison of experimental and calculated anharmonic spectra of Na^+18C6. The

structures are the same as those shown in Figure S13. The relative zero-point corrected energies and Gibbs free energies (kJ mol^{-1}) at 120 K/298 K are given for each conformer.

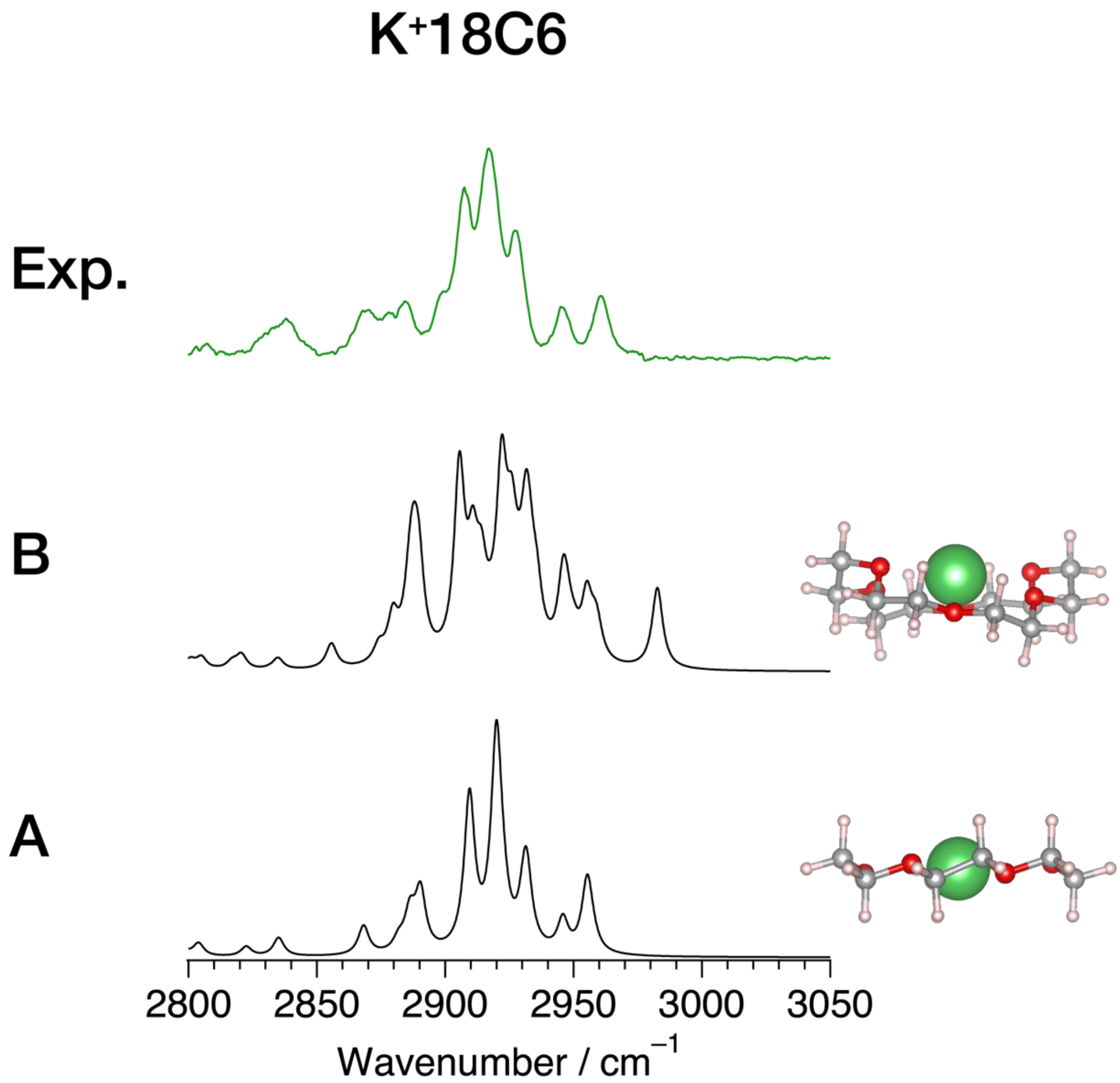

Figure S29. Comparison of experimental and calculated anharmonic spectra of K^+18C6. The structures are the same as those shown in Figure S14. The relative zero-point corrected energies and Gibbs free energies (kJ mol^{-1}) at 120 K/298 K are given for each conformer.

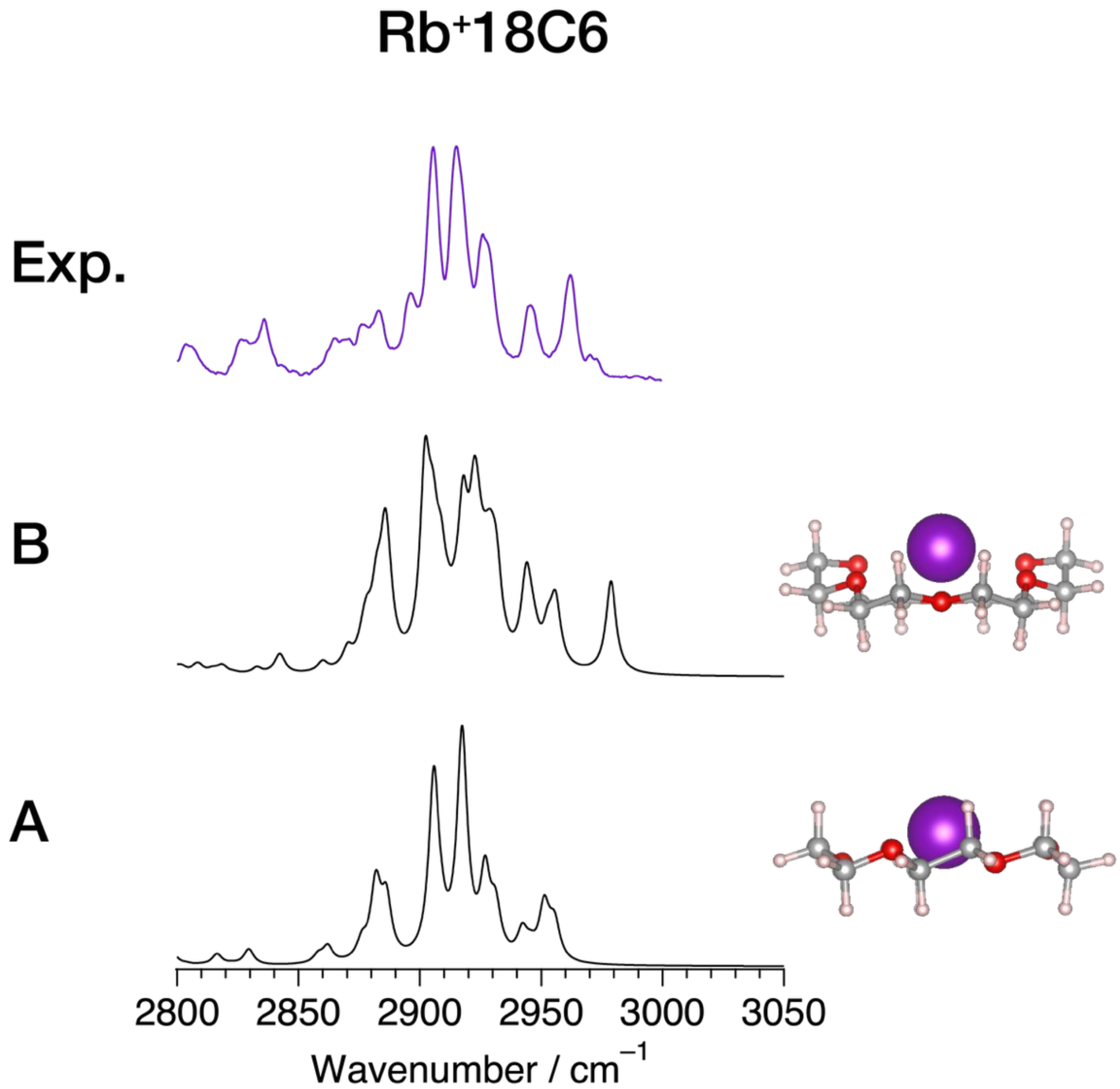

Figure S30. Comparison of experimental and calculated anharmonic spectra of Rb^+18C6. The structures are the same as those shown in Figure S15. The relative zero-point corrected energies and Gibbs free energies (kJ mol^{-1}) at 120 K/298 K are given for each conformer.

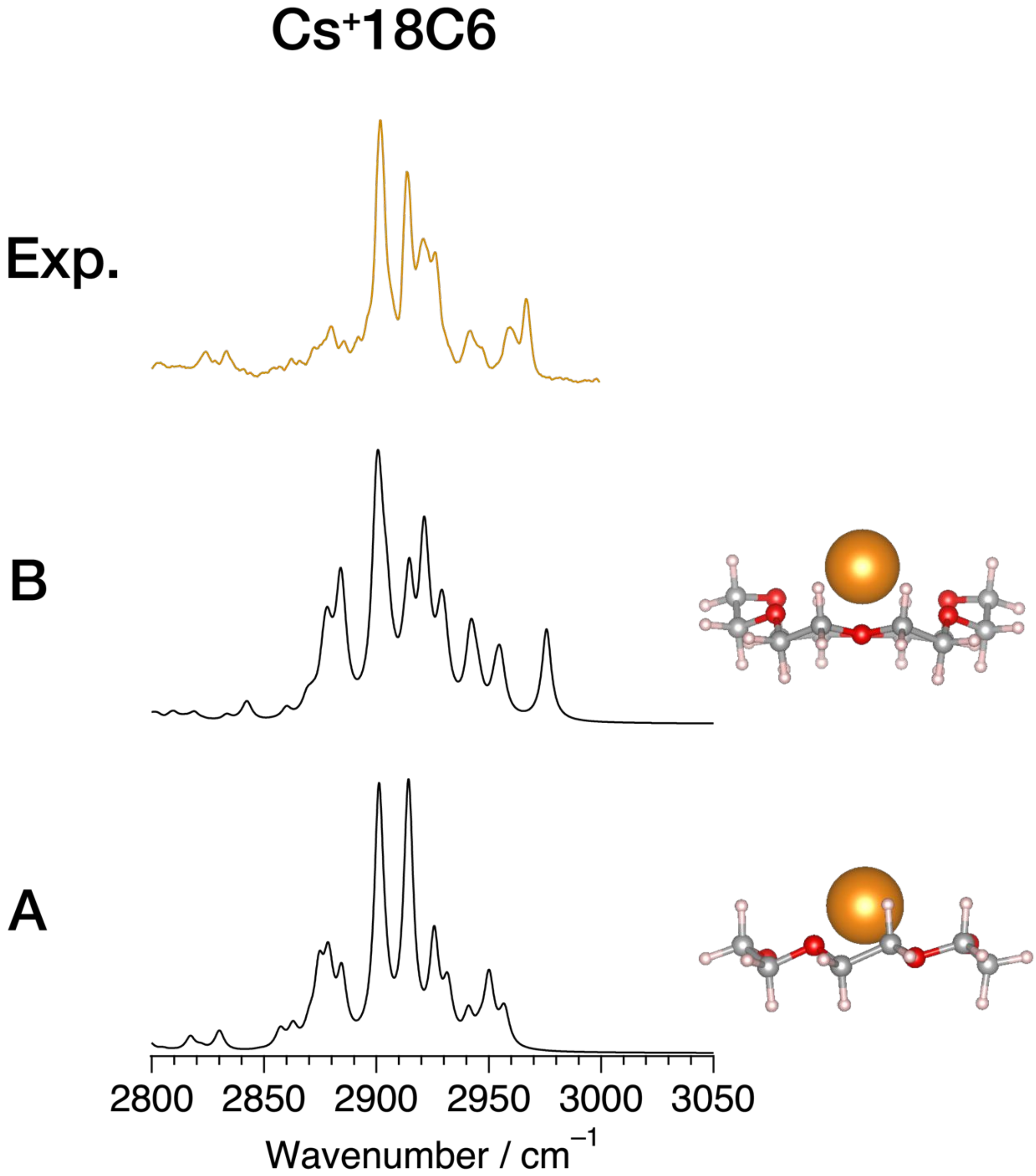

Figure S31. Comparison of experimental and calculated anharmonic spectra of Cs^+18C6. The structures are the same as those shown in Figure S16. The relative zero-point corrected energies and Gibbs free energies (kJ mol^{-1}) at 120 K/298 K are given for each conformer.

Table S2: Atomic coordinates (in Å) of the lowest energy structure of Li^+12C4, computed at the B3LYP/6-311++G(d,p) level.

H	1.380312000	2.955004000	0.872876000
H	1.267560000	2.655136000	-0.880776000
H	3.295481000	1.507866000	-0.010316000
H	2.324949000	0.726943000	1.263912000
H	3.279410000	-1.299665000	0.259748000
H	2.459077000	-1.848820000	-1.210511000
H	1.435804000	-2.987352000	0.726252000
H	1.136552000	-1.433476000	1.541118000
H	-1.136628000	-1.433388000	1.541121000
H	-1.435976000	-2.987255000	0.726278000
H	-2.459229000	-1.848703000	-1.210477000
H	-3.279498000	-1.299477000	0.259791000
H	-2.324874000	0.727074000	1.263914000
H	-3.295382000	1.508059000	-0.010292000
H	-1.267377000	2.655180000	-0.880809000
H	-1.380111000	2.955084000	0.872835000
Li	0.000017000	-0.008401000	-0.965291000
C	1.240541000	2.183136000	0.109278000
C	2.308683000	1.107518000	0.238410000
C	2.328855000	-1.293816000	-0.280270000
C	1.225726000	-1.924790000	0.564986000
C	-1.225840000	-1.924708000	0.564996000
C	-2.328956000	-1.293690000	-0.280249000
C	-2.308605000	1.107646000	0.238410000
C	-1.240388000	2.183193000	0.109252000
O	1.967333000	0.045144000	-0.676774000
O	-0.000053000	-1.753726000	-0.165592000
O	-1.967365000	0.045243000	-0.676779000
O	0.000059000	1.489097000	0.291936000

Table S3: Atomic coordinates (in Å) of the lowest energy structure of Na^{+}12C4, computed at the B3LYP/6-311++G(d,p) level.

H	-0.908456000	1.381789000	1.575689000
H	-1.893959000	2.704756000	0.923753000
H	0.528102000	3.297260000	0.848754000
H	-0.165235000	3.211282000	-0.777063000
H	0.165256000	-3.211298000	-0.776990000
H	-0.528124000	-3.297242000	0.848810000
H	1.893934000	-2.704736000	0.923861000
H	0.908414000	-1.381755000	1.575742000
H	3.211300000	0.165236000	-0.776997000
H	3.297250000	-0.528102000	0.848820000
H	2.704742000	1.893958000	0.923808000
H	1.381767000	0.908454000	1.575724000
H	-2.704767000	-1.893939000	0.923776000
H	-1.381810000	-0.908421000	1.575707000
H	-3.297272000	0.528120000	0.848722000
H	-3.211279000	-0.165252000	-0.777079000
C	-1.945097000	-1.146664000	0.665872000
C	-2.621481000	0.095948000	0.103645000
C	-1.146680000	1.945088000	0.665855000
C	0.095945000	2.621476000	0.103662000
C	-0.095947000	-2.621474000	0.103715000
C	1.146662000	-1.945073000	0.665927000
C	2.621478000	-0.095945000	0.103717000
C	1.945079000	1.146678000	0.665899000
O	-1.067492000	-1.663090000	-0.343066000
O	-1.663086000	1.067496000	-0.343104000
O	1.067501000	1.663083000	-0.343073000
O	1.663095000	-1.067503000	-0.343038000
Na	0.000021000	-0.000017000	-1.616049000

Table S4: Atomic coordinates (in Å) of the lowest energy structure of K^{+}12C4, computed at the B3LYP/6-311++G(d,p) level.

H	-2.449853000	2.090662000	0.558216000
H	-2.932808000	1.589054000	-1.066513000

H	-0.999074000	3.120924000	-1.170723000
H	-0.529203000	1.519662000	-1.765808000
H	0.529233000	-1.519370000	-1.766048000
H	0.999094000	-3.120731000	-1.171220000
H	2.932827000	-1.588879000	-1.066722000
H	2.449843000	-2.090754000	0.557916000
H	1.519532000	0.529363000	-1.765871000
H	3.120838000	0.999181000	-1.170862000
H	1.588975000	2.932905000	-1.066362000
H	2.090702000	2.449801000	0.558286000
H	-1.588957000	-2.932730000	-1.066871000
H	-2.090712000	-2.449894000	0.557847000
H	-3.120819000	-0.998989000	-1.171079000
H	-1.519503000	-0.529073000	-1.765985000
C	-1.511915000	-2.139474000	-0.314776000
C	-2.075993000	-0.841622000	-0.874974000
C	-2.139497000	1.511943000	-0.314482000
C	-0.841686000	2.076071000	-0.874724000
C	0.841700000	-2.075927000	-0.875051000
C	2.139502000	-1.511892000	-0.314693000
C	2.076008000	0.841765000	-0.874800000
C	1.511920000	2.139524000	-0.314398000
O	-0.161407000	-1.997195000	0.141345000
O	-1.997186000	0.161394000	0.141507000
O	0.161404000	1.997170000	0.141676000
O	1.997184000	-0.161417000	0.141515000
K	-0.000016000	-0.000162000	1.989518000

Table S5: Atomic coordinates (in Å) of the lowest energy structure of Rb^{+}12C4, computed at the B3LYP/6-311++G(d,p) level.

H	-2.064853000	-2.473761000	0.187348000
H	-1.558881000	-2.948249000	-1.437667000
H	-3.093518000	-1.038131000	-1.559833000
H	-1.490638000	-0.538327000	-2.120849000
H	1.490287000	0.538360000	-2.121086000

H	3.093261000	1.038156000	-1.560328000
H	1.558645000	2.948273000	-1.437877000
H	2.064884000	2.473757000	0.187047000
H	-0.538517000	1.490477000	-2.120912000
H	-1.038271000	3.093401000	-1.559972000
H	-2.948379000	1.558775000	-1.437518000
H	-2.473743000	2.064868000	0.187417000
H	2.948143000	-1.558752000	-1.438026000
H	2.473775000	-2.064869000	0.186979000
H	1.038016000	-3.093378000	-1.560188000
H	0.538169000	-1.490447000	-2.121024000
C	2.157315000	-1.483615000	-0.682149000
C	0.862845000	-2.057491000	-1.241160000
C	-1.483676000	-2.157365000	-0.681855000
C	-2.057603000	-0.862937000	-1.240911000
C	2.057398000	0.862957000	-1.241236000
C	1.483564000	2.157376000	-0.682065000
C	-0.863048000	2.057510000	-1.240987000
C	-2.157427000	1.483626000	-0.681771000
O	2.017548000	-0.136501000	-0.222059000
O	-0.136521000	-2.017564000	-0.221896000
O	-2.017584000	0.136505000	-0.221725000
O	0.136485000	2.017568000	-0.221888000
Rb	0.000162000	-0.000016000	1.972428000

Table S6: Atomic coordinates (in Å) of the lowest energy structure of Cs^{+}12C4, computed at the B3LYP/6-311++G(d,p) level.

H	0.157017000	-1.054044000	3.043910000
H	1.781887000	-0.413180000	3.309098000
H	1.913293000	-2.516152000	2.066835000
H	2.459436000	-1.194245000	1.025105000
H	2.459434000	1.194248000	-1.025105000
H	1.913288000	2.516154000	-2.066836000
H	1.781885000	0.413182000	-3.309099000
H	0.157015000	1.054044000	-3.043910000

H	2.459436000	-1.025103000	-1.194247000
H	1.913292000	-2.066834000	-2.516153000
H	1.781888000	-3.309097000	-0.413181000
H	0.157018000	-3.043910000	-1.054044000
H	1.781884000	3.309100000	0.413181000
H	0.157014000	3.043910000	1.054044000
H	1.913289000	2.066837000	2.516153000
H	2.459435000	1.025107000	1.194247000
C	1.025013000	2.542421000	0.619699000
C	1.584236000	1.534089000	1.613788000
C	1.025016000	-0.619699000	2.542421000
C	1.584238000	-1.613787000	1.534088000
C	1.584235000	1.613789000	-1.534088000
C	1.025014000	0.619700000	-2.542421000
C	1.584237000	-1.534087000	-1.613788000
C	1.025017000	-2.542420000	-0.619699000
O	0.563905000	1.941465000	-0.592151000
O	0.563907000	0.592151000	1.941465000
O	0.563908000	-1.941465000	0.592151000
O	0.563907000	-0.592151000	-1.941465000
Cs	-1.925701000	-0.000001000	0.000000000

Table S7: Atomic coordinates (in Å) of the lowest energy structure of Li^+15C5, computed at the B3LYP/6-311++G(d,p) level.

H	3.546363000	1.754999000	-0.258513000
H	3.007602000	0.683354000	1.056055000
H	2.799689000	0.170195000	-1.972866000
H	3.838113000	-0.721202000	-0.843448000
H	2.330396000	-1.677665000	1.181432000
H	2.728254000	-2.817946000	-0.129719000
H	0.729749000	-3.567770000	1.185316000
H	0.326850000	-3.238353000	-0.520891000
H	-1.763228000	-3.257243000	0.966225000
H	-2.158697000	-1.661804000	1.615052000
H	-1.797115000	-2.347088000	-1.369668000
H	-3.335889000	-1.975600000	-0.580133000

H	1.826753000	3.435646000	0.572603000
H	1.180053000	2.301282000	1.787363000
H	-0.262820000	3.403828000	-0.690137000
H	-0.671344000	3.746898000	1.012492000
H	-2.969126000	2.622116000	0.161909000
H	-2.140578000	2.209882000	-1.362370000
H	-3.852451000	0.496134000	-0.872359000
H	-3.172578000	0.253699000	0.756978000
Li	-0.006336000	0.047178000	-0.188997000
C	2.814685000	0.987557000	0.019329000
C	2.889102000	-0.186196000	-0.945526000
C	1.983050000	-2.100510000	0.231286000
C	0.651000000	-2.785569000	0.422773000
C	-1.662778000	-2.181239000	0.792893000
C	-2.257794000	-1.786172000	-0.554246000
C	1.138362000	2.598676000	0.732280000
C	-0.266671000	2.994488000	0.326838000
C	-2.309819000	1.894901000	-0.325376000
C	-2.932535000	0.518358000	-0.278506000
O	1.482113000	1.488070000	-0.099938000
O	1.775503000	-1.066568000	-0.738139000
O	-0.283857000	-1.779617000	0.841418000
O	-1.973198000	-0.403431000	-0.819181000
O	-1.056886000	1.801462000	0.358490000

Table S8: Atomic coordinates (in Å) of the lowest energy structure of Na^+15C5, computed at the B3LYP/6-311++G(d,p) level.

H	-2.695358000	-1.652655000	-1.387913000
H	-3.374478000	-2.332363000	0.110026000
H	-4.163350000	-0.001471000	-0.240957000
H	-3.197354000	-0.160832000	1.248142000
H	-1.308694000	-3.830288000	0.444684000
H	-0.746258000	-3.219815000	-1.131709000
H	-2.373420000	1.922532000	1.397728000
H	-3.248512000	2.574502000	-0.010896000

H	1.169419000	-3.910684000	0.310145000
H	0.632013000	-2.775428000	1.575116000
H	-1.182647000	3.858346000	0.453624000
H	-1.096281000	3.078187000	-1.143905000
H	1.334276000	3.154648000	-0.963502000
H	1.407935000	3.700303000	0.732305000
H	2.447425000	-1.222169000	1.455800000
H	3.297251000	-2.354711000	0.379488000
H	4.098220000	-0.020733000	0.009817000
H	3.341491000	-0.702527000	-1.440599000
H	3.371353000	2.304165000	0.175003000
H	2.362150000	1.501131000	1.405239000
C	2.592899000	-1.513867000	0.406704000
C	3.151992000	-0.364018000	-0.419879000
C	2.400974000	1.829293000	0.359211000
C	1.291290000	2.825195000	0.082145000
C	-1.125327000	2.892443000	-0.062269000
C	-2.335871000	2.058576000	0.310250000
C	0.656162000	-2.959677000	0.493961000
C	-0.757973000	-3.022700000	-0.051873000
C	-2.694086000	-1.572621000	-0.292631000
C	-3.159580000	-0.199005000	0.153109000
O	1.336417000	-1.886937000	-0.166245000
O	2.215010000	0.718455000	-0.526694000
O	0.044192000	2.172609000	0.338729000
O	-2.229266000	0.779605000	-0.325872000
O	-1.369312000	-1.757442000	0.211821000
Na	-0.015854000	0.003439000	-0.500356000

Table S9: Atomic coordinates (in Å) of the lowest energy structure of K^+15C5, computed at the B3LYP/6-311++G(d,p) level.

H	-0.504537000	3.454973000	-0.542519000
H	-0.253048000	3.666722000	1.205888000
H	2.099074000	3.159236000	0.999981000
H	2.028645000	2.815330000	-0.742826000

H	-2.577416000	3.025924000	0.793765000
H	-1.726103000	1.731972000	1.673403000
H	2.637757000	0.807708000	1.485960000
H	3.847289000	1.538531000	0.403315000
H	-3.660999000	0.268796000	-1.057131000
H	-3.954418000	0.979546000	0.537333000
H	3.147640000	-1.608226000	-1.162695000
H	4.045295000	-1.022249000	0.243387000
H	-2.264527000	-0.634430000	1.518531000
H	-3.667683000	-1.431481000	0.773367000
H	2.583455000	-2.868198000	0.908892000
H	1.999849000	-1.379346000	1.678733000
H	-0.268500000	-2.060038000	1.814382000
H	0.197622000	-3.661401000	1.204921000
H	-2.171210000	-3.295011000	0.712033000
H	-1.239029000	-3.368475000	-0.790742000
C	-0.466555000	2.929539000	0.421489000
C	1.877460000	2.383485000	0.256146000
C	-1.792960000	2.262938000	0.716356000
C	2.804316000	1.207324000	0.479649000
C	-3.205563000	0.480102000	-0.087234000
C	3.047513000	-1.093044000	-0.204839000
C	-2.777084000	-0.823817000	0.566719000
C	2.131195000	-1.886654000	0.713970000
C	-0.125742000	-2.656251000	0.902892000
C	-1.416893000	-2.776215000	0.109592000
O	0.537186000	1.916067000	0.399932000
O	-2.098308000	1.352559000	-0.343459000
O	2.534317000	0.208465000	-0.508357000
O	0.862964000	-2.041317000	0.076324000
O	-1.915270000	-1.512774000	-0.342803000
K	-0.006336000	0.017456000	-1.534711000

Table S10: Atomic coordinates (in Å) of the lowest energy structure of Rb^+15C5, computed at the B3LYP/6-311++G(d,p) level.

H	3.705335000	-0.261981000	-0.733726000
H	3.954913000	-0.963728000	0.870729000
H	3.633357000	1.421313000	1.125693000
H	2.186051000	0.621158000	1.775140000
H	1.239963000	3.372186000	-0.527699000
H	2.162351000	3.306094000	0.980208000
H	-0.182386000	3.637524000	1.493064000
H	0.271877000	2.016017000	2.051761000
H	-2.090210000	-3.150699000	1.269604000
H	-2.039090000	-2.804121000	-0.471727000
H	-2.622375000	-0.794693000	1.759655000
H	-3.852627000	-1.549581000	0.719074000
H	-4.053603000	1.038283000	0.566013000
H	-3.187878000	1.623448000	-0.858671000
H	-1.955850000	1.343476000	1.940369000
H	-2.575135000	2.845474000	1.229056000
H	0.242024000	-3.650399000	1.470976000
H	0.510530000	-3.441118000	-0.273616000
H	1.701535000	-1.698302000	1.939847000
H	2.561595000	-3.022524000	1.117306000
C	0.462499000	-2.911994000	0.688883000
C	1.789240000	-2.251966000	0.997650000
C	3.218195000	-0.479013000	0.219685000
C	2.753449000	0.821036000	0.857568000
C	1.421693000	2.775770000	0.369459000
C	0.128901000	2.638360000	1.157964000
C	-1.877357000	-2.371304000	0.526184000
C	-2.812151000	-1.203312000	0.761383000
C	-3.066926000	1.096557000	0.090773000
C	-2.120135000	1.873547000	0.993009000
O	-0.539744000	-1.900499000	0.658015000
O	2.139516000	-1.372647000	-0.072804000
O	1.951056000	1.529181000	-0.088514000
O	-0.873386000	2.058401000	0.325127000

O	-2.587189000	-0.209091000	-0.240679000
Rb	0.007653000	-0.017748000	-1.677502000

Table S11: Atomic coordinates (in Å) of the lowest energy structure of Cs^{+}15C5, computed at the B3LYP/6-311++G(d,p) level.

H	-2.028606000	2.796503000	-0.180273000
H	-2.080456000	3.141166000	1.560637000
H	-3.852854000	1.560031000	1.020777000
H	-2.622012000	0.783114000	2.043223000
H	-3.210727000	-1.624006000	-0.570499000
H	-4.063069000	-1.042443000	0.862941000
H	-2.580213000	-2.835664000	1.521150000
H	-1.941961000	-1.331290000	2.209045000
H	3.956285000	0.934410000	1.195213000
H	3.726822000	0.247717000	-0.418003000
H	2.145268000	-0.634991000	2.050048000
H	3.607574000	-1.439124000	1.441592000
H	2.144501000	-3.325268000	1.242856000
H	1.227101000	-3.373973000	-0.268124000
H	0.264045000	-2.009698000	2.307412000
H	-0.188854000	-3.635715000	1.763382000
H	1.692148000	1.659494000	2.234338000
H	2.558377000	3.001651000	1.449285000
H	0.243776000	3.627100000	1.772921000
H	0.520689000	3.423594000	0.029706000
C	1.791621000	2.229578000	1.303247000
C	0.466259000	2.889821000	0.989785000
C	-1.870285000	2.360408000	0.817283000
C	-2.816035000	1.200697000	1.049597000
C	-3.081019000	-1.094870000	0.376723000
C	-2.122481000	-1.868679000	1.268749000
C	3.224522000	0.463606000	0.527943000
C	2.736887000	-0.836338000	1.148461000
C	1.412346000	-2.782629000	0.631827000
C	0.119256000	-2.638461000	1.418395000

O	2.163717000	1.372381000	0.223459000
O	-0.537389000	1.882195000	0.951463000
O	-2.614001000	0.212146000	0.038437000
O	-0.887189000	-2.067885000	0.586223000
O	1.960154000	-1.542611000	0.181401000
Cs	0.011517000	0.030705000	-1.751046000

Table S12: Atomic coordinates (in Å) of the lowest energy structure of Li^+18C6, computed at the B3LYP/6-311++G(d,p) level.

H	-1.599801000	-0.671610000	2.611091000
H	-3.341943000	1.633479000	-0.263515000
H	-3.627894000	0.295834000	-1.393737000
H	-1.601100000	0.669493000	-2.611079000
H	-2.269511000	2.295779000	-2.376066000
H	-3.627209000	-0.300653000	1.394035000
H	-3.339811000	-1.638095000	0.263948000
H	-2.266052000	-2.298813000	2.376275000
H	-0.669972000	3.818558000	-1.045616000
H	-1.505917000	2.917535000	0.244593000
H	1.503061000	2.917581000	-0.244662000
H	0.667179000	3.819225000	1.045151000
H	-1.501938000	-2.919349000	-0.244750000
H	-0.664877000	-3.819474000	1.045365000
H	0.672250000	-3.818289000	-1.045414000
H	1.507050000	-2.915737000	0.244467000
H	1.598584000	0.669989000	2.611268000
H	2.265859000	2.297003000	2.377994000
H	3.626333000	0.298748000	1.394872000
H	3.340235000	1.637163000	0.265651000
H	1.599835000	-0.667862000	-2.611286000
H	2.269238000	-2.293978000	-2.377825000
H	3.342286000	-1.632603000	-0.265223000
H	3.627009000	-0.294012000	-1.394591000
Li	-0.001083000	0.000003000	-0.000205000
C	-2.850565000	0.854077000	-0.858242000

C	-1.848967000	1.423679000	-1.862943000
C	-2.849288000	-0.857988000	0.858456000
C	-1.846759000	-1.426176000	1.863038000
C	-0.632109000	2.922499000	-0.415262000
C	0.629351000	2.922794000	0.415313000
C	-0.628122000	-2.923303000	0.415102000
C	0.633328000	-2.921969000	-0.415488000
C	1.846328000	1.424976000	1.863906000
C	2.848957000	0.856926000	0.859372000
C	1.848486000	-1.422476000	-1.863847000
C	2.850194000	-0.853055000	-0.859178000
O	-2.101170000	-0.001540000	-0.000037000
O	-0.606999000	1.745196000	-1.231615000
O	0.604408000	1.745946000	1.232241000
O	-0.604446000	-1.746050000	1.231564000
O	0.606926000	-1.745076000	-1.232310000
O	2.100701000	0.001546000	-0.000055000

Table S13: Atomic coordinates (in Å) of the lowest energy structure of Na^+18C6, computed at the B3LYP/6-311++G(d,p) level.

H	-3.228822000	1.404236000	0.644811000
H	-4.144450000	0.946391000	-0.812699000
H	-4.157418000	-0.892172000	0.813823000
H	-3.247951000	-1.362593000	-0.643551000
H	2.858545000	2.776716000	1.667236000
H	2.710215000	2.570906000	-0.095718000
H	-2.867176000	-2.731388000	1.715335000
H	-1.298676000	-2.221562000	2.365636000
H	4.128510000	0.736215000	0.977400000
H	3.176929000	-0.696396000	1.382468000
H	-1.269098000	2.234321000	-2.366762000
H	-2.830941000	2.765244000	-1.717285000
H	3.186845000	0.656078000	-1.381220000

H	4.119193000	-0.788891000	-0.975269000
H	0.940187000	-4.120279000	-0.728222000
H	0.345675000	-2.889460000	-1.869680000
H	-1.778926000	-3.174773000	-0.517347000
H	-1.017727000	-4.111266000	0.786263000
H	0.381773000	2.886626000	1.867812000
H	0.993563000	4.108845000	0.726300000
H	-0.964158000	4.123336000	-0.790791000
H	-1.736878000	3.198683000	0.514495000
H	2.675754000	-2.605060000	0.096629000
H	2.823563000	-2.812235000	-1.666204000
C	-3.239706000	0.740263000	-0.227691000
C	-3.249792000	-0.698610000	0.228986000
C	2.228347000	2.368457000	0.867846000
C	-1.906496000	-2.270118000	1.460087000
C	3.213952000	0.182709000	0.735464000
C	-1.876178000	2.292289000	-1.461269000
C	3.211805000	-0.223367000	-0.734110000
C	0.831555000	-3.041944000	-0.897097000
C	-1.189471000	-3.096131000	0.405595000
C	0.870641000	3.032105000	0.895629000
C	-1.148585000	3.111069000	-0.408387000
C	2.197745000	-2.396036000	-0.867462000
O	2.052010000	0.960581000	1.051932000
O	-2.089759000	-0.915629000	1.036309000
O	2.039960000	-0.985930000	-1.051318000
O	0.083842000	2.445036000	-0.140575000
O	-2.076600000	0.941067000	-1.034790000
O	0.051279000	-2.445270000	0.138656000
Na	0.004791000	-0.000407000	0.001751000

Table S14: Atomic coordinates (in Å) of the lowest energy structure of K^{+}18C6, computed at the B3LYP/6-311++G(d,p) level.

H	-3.462620000	3.117645000	0.004016000
H	2.185191000	2.893921000	-1.398297000

H	1.066407000	3.465815000	1.398381000
H	0.968616000	4.557517000	-0.003980000
H	3.127257000	3.454000000	0.004168000
H	4.554897000	-0.981411000	-0.003988000
H	4.431378000	1.439903000	0.004343000
H	3.535174000	0.809550000	-1.398277000
H	3.598719000	-0.445452000	1.398321000
H	2.468324000	-2.656348000	1.398310000
H	3.462619000	-3.117647000	-0.004059000
H	1.413664000	-3.339577000	-1.398277000
H	1.427629000	-4.435271000	0.004297000
H	-2.185176000	-2.893920000	1.398308000
H	-1.066422000	-3.465812000	-1.398382000
H	-3.127258000	-3.454001000	-0.004146000
H	-0.968618000	-4.557517000	0.003976000
H	-3.535149000	-0.809540000	1.398305000
H	-4.431378000	-1.439904000	-0.004293000
H	-3.598744000	0.445441000	-1.398300000
H	-4.554899000	0.981410000	0.004021000
H	-2.468313000	2.656320000	-1.398337000
H	-1.427631000	4.435271000	-0.004350000
H	-1.413677000	3.339604000	1.398246000
C	-2.527752000	2.629999000	-0.302149000
C	-1.365256000	3.382766000	0.302038000
C	-1.013793000	-3.504078000	-0.302190000
C	1.013790000	3.504078000	0.302188000
C	1.365252000	-3.382760000	-0.302070000
C	-3.612191000	0.509063000	-0.302106000
C	2.527754000	-2.630006000	0.302122000
C	3.541715000	0.874098000	-0.302074000
C	2.246924000	2.873695000	-0.302103000
C	-2.246922000	-2.873695000	0.302114000
C	-3.541710000	-0.874096000	0.302103000
C	3.612185000	-0.509066000	0.302127000
O	0.143290000	-2.807488000	0.157675000
O	-0.143289000	2.807486000	-0.157685000

O	2.503042000	-1.279662000	-0.157765000
O	-2.359794000	-1.527802000	-0.157581000
O	-2.503042000	1.279663000	0.157763000
O	2.359791000	1.527801000	0.157592000
K	0.000002000	0.000001000	0.000001000

Table S15: Atomic coordinates (in Å) of the lowest energy structure of Rb^{+}18C6, computed at the B3LYP/6-311++G(d,p) level.

H	3.463080000	-0.572950000	1.581036000
H	-1.234916000	3.286273000	1.580745000
H	1.136747000	3.323894000	1.578813000
H	-3.445099000	-0.677844000	1.580628000
H	2.309944000	-2.645095000	1.579773000
H	-2.229217000	-2.714036000	1.579602000
H	-1.226514000	-3.523552000	-1.201019000
H	3.666574000	0.699596000	-1.199454000
H	-3.686226000	0.588884000	-1.199628000
H	1.332636000	-3.484727000	-1.200934000
H	2.350976000	2.894842000	-1.202147000
H	-2.440635000	2.825961000	-1.199047000
H	4.560258000	-1.113814000	0.287209000
H	-1.314717000	4.505569000	0.285726000
H	1.178176000	4.543461000	0.282226000
H	-4.524437000	-1.251195000	0.285818000
H	3.345732000	-3.292577000	0.284409000
H	-3.244639000	-3.392185000	0.283771000
H	-1.125227000	-4.523404000	0.264941000
H	4.480971000	1.287302000	0.266995000
H	-4.517255000	1.151465000	0.267346000
H	1.261577000	-4.487256000	0.264999000
H	3.255612000	3.336866000	0.262592000
H	-3.354969000	3.236278000	0.268774000
C	3.591743000	-0.638539000	0.492959000
C	-1.242436000	3.429346000	0.492515000
C	1.138557000	3.465817000	0.490408000

C	-3.570863000	-0.746960000	0.492427000
C	2.432481000	-2.718689000	0.491499000
C	-2.349157000	-2.791089000	0.491281000
C	-1.145408000	-3.489862000	-0.105353000
C	3.596110000	0.753226000	-0.103853000
C	-3.616979000	0.644210000	-0.104038000
C	1.250503000	-3.453571000	-0.105272000
C	2.365918000	2.809572000	-0.106380000
C	-2.450398000	2.737348000	-0.103491000
O	2.534785000	-1.412198000	-0.075330000
O	-0.044048000	2.899897000	-0.075215000
O	-2.490586000	-1.488136000	-0.075266000
O	0.042403000	-2.803390000	0.284976000
O	2.407592000	1.438842000	0.285623000
O	-2.449305000	1.364988000	0.285109000
Rb	-0.000151000	-0.000179000	-0.663745000

Table S16: Atomic coordinates (in Å) of the lowest energy structure of Cs^+18C6, computed at the B3LYP/6-311++G(d,p) level.

H	3.367295000	-0.667602000	-1.838406000
H	4.528781000	-1.239384000	-0.615666000
H	3.748149000	0.582956000	0.931868000
H	4.494766000	1.157587000	-0.573155000
H	3.355113000	3.206390000	-0.573488000
H	2.472650000	2.875363000	0.931325000
H	1.337099000	4.499772000	-0.617034000
H	1.209646000	3.210902000	-1.839201000
H	-1.105349000	3.249923000	-1.838582000
H	-1.190787000	4.541151000	-0.615263000
H	-2.378419000	2.953363000	0.931805000
H	-3.249522000	3.313684000	-0.572768000
H	-4.454974000	1.302200000	-0.572930000
H	-3.726190000	0.703877000	0.931615000
H	-4.566319000	-1.092240000	-0.615950000
H	-3.386872000	-0.558492000	-1.838709000

H	-2.261078000	-2.581257000	-1.838895000
H	-3.337242000	-3.301987000	-0.616819000
H	-1.369329000	-3.536907000	0.931521000
H	-1.244402000	-4.470630000	-0.573599000
H	1.099342000	-4.508323000	-0.574580000
H	1.254545000	-3.579910000	0.930956000
H	3.228677000	-3.407766000	-0.617615000
H	2.176056000	-2.652132000	-1.839107000
C	-2.425352000	-2.706298000	-0.761080000
C	3.557181000	-0.747255000	-0.760487000
C	2.336445000	-2.782909000	-0.761383000
C	1.242306000	3.414701000	-0.761377000
C	-3.579272000	-0.631869000	-0.760794000
C	-1.131152000	3.453713000	-0.760573000
C	-2.363955000	2.812714000	-0.159113000
C	-1.254067000	-3.453519000	-0.159265000
C	2.453797000	2.734642000	-0.159500000
C	-3.595589000	0.757512000	-0.159320000
C	1.141760000	-3.492249000	-0.159749000
C	3.618592000	0.640802000	-0.158973000
O	-2.570271000	-1.428844000	-0.142502000
O	2.522764000	-1.511056000	-0.142272000
O	0.047678000	2.939611000	-0.142815000
O	-2.382193000	1.427014000	-0.487808000
O	-0.044964000	-2.775685000	-0.486479000
O	2.427128000	1.348802000	-0.486928000
Cs	-0.000126000	0.000015000	1.105869000
